# Glucose-Responsive Nanomedicine in Diabetes Therapy: Emerging Advances and Clinical Prospects

**DOI:** 10.3390/jfb17090424

**Published:** 2026-08-22

**Authors:** Adnan Alsaei, Ayah Binrajab, Shahd Alsaei, Fatema Rahimi, Ahmad Zarwi, Helen N. Zarwi, Renad Alansari, G. Roshan Deen

**Affiliations:** 1 Materials for Medicine Research Group, School of Medicine, Royal College of Surgeons in Ireland (RCSI), Medical University of Bahrain, Busaiteen 228, Bahrain; 23200222@rcsi.com (A.A.); 24200305@rcsi.com (A.B.); 23200122@rcsi.com (F.R.); 24204621@rcsi.com (A.Z.); 23200769@rcsi.com (H.N.Z.); 21200799@rcsi.com (R.A.); 2 School of Population Health, RCSI University of Medicine and Health Sciences, 118 St Stephen’s Green, Dublin 2, D02 YN77 Dublin, Ireland; shahdalsaei25@rcsi.ie

**Keywords:** glucose-responsive nanomedicine, diabetes mellitus, insulin delivery, glucose oxidase, phenylboronic acid, closed-loop drug delivery

## Abstract

Diabetes mellitus continues to impose a substantial global health burden, underscoring the need for therapeutic systems capable of achieving precise, adaptive, and patient-friendly glycemic control. Conventional diabetes treatments, including repeated insulin injections and oral hypoglycemic agents, are often constrained by non-physiological drug release, poor adherence, systemic side effects, and the persistent risk of hypoglycemia. In this context, glucose-responsive nanomedicine has emerged as a promising platform for next-generation diabetes therapy by enabling self-regulated and glucose-triggered delivery of insulin and other antidiabetic agents. This review highlights recent advances in glucose-responsive nanomedicine, focusing on the principal sensing mechanisms, including glucose oxidase-based, phenylboronic acid-based, and lectin-mediated systems, as well as the nanoscale carriers engineered to support them, such as polymeric nanoparticles, nanogels, micelles, liposomes, and hybrid nanostructures. These smart platforms offer significant potential to improve drug stability, enhance targeting efficiency, reduce dosing frequency, and more closely mimic endogenous insulin secretion. The review further examines their emerging role in precision diabetes care, particularly in combination with continuous glucose monitoring technologies, wearable devices, and closed-loop therapeutic systems. Despite notable progress at the preclinical level, important barriers to clinical translation remain, including challenges related to biocompatibility, long-term safety, reproducibility, scalable manufacturing, and regulatory approval. Collectively, glucose-responsive nanomedicine represents a rapidly advancing and clinically relevant field with the potential to redefine diabetes management through intelligent and personalized therapeutic strategies. This review provides a focused overview of current developments, key translational challenges, and future directions toward clinical implementation.

## 1. Introduction

Diabetes mellitus (DM) is a group of chronic metabolic disorders characterized by persistent hyperglycemia resulting from defects in insulin secretion, insulin action, or both [1]. Type 1 diabetes mellitus (T1DM) accounts for ~10% of the diabetic patient population; it is primarily caused by autoimmune damage to the insulin-producing β-cells of the pancreatic islets, leading to insulin deficiency [2,3]. In contrast, type 2 diabetes mellitus (T2DM), the most prevalent form of diabetes worldwide, is marked by insulin resistance (IR) and a progressive decline in β-cell function and insulin secretion [4]. In both forms, DM contributes to serious complications due to prolonged poor glycemic control, such as diabetic retinopathy, nephropathy, neuropathy, cardiovascular disease, and stroke [3,5].

Diabetes is a major global health challenge because of its rapidly increasing prevalence. According to the latest World Health Organization (WHO) report, the global number of adults living with diabetes increased significantly from 200 million in 1990 to 830 million in 2022, while the global prevalence doubled from 7% to 14% over the same period [6,7]. Empirical evidence has demonstrated that only 55.8% of people living with DM had been diagnosed in 2023, while only 21.1% were receiving the required diabetes management medication [8]. Moreover, the burden of diabetes and the coverage of diabetes medication are unequally distributed, with low- and middle-income countries (LMICs) being particularly affected [6]. Critically, the global challenges of DM are not limited to its rising prevalence; persistent gaps in early diagnosis, access to treatment, and continuity of long-term care influence population health outcomes, resulting in increased disability and premature mortality. For instance, the WHO reported that diabetes directly caused 1.6 million deaths in 2021, while disability-adjusted life years (DALYs) more than doubled between 2000 and 2021 [8].

Moreover, health-related quality of life (HRQoL) is profoundly affected by diabetes, with lifelong self-management, insulin use, frequent glucose monitoring, fear of hypoglycemia, and the risk of complications often reducing physical functioning, emotional well-being, and everyday participation [9]. Accordingly, HRQoL represents a significant outcome in diabetes care, complementing the clinical assessment of glycemic control [10]. Thus, enhancing diabetes therapy is crucial not only for achieving glycemic control but also for reducing disability, premature mortality, treatment burden, and the overall population health impact of the disease [11].

Conventional diabetes treatments include oral glucose-lowering agents and exogenous insulin therapy. These treatments remain fundamental for managing hyperglycemia and delaying disease complications. However, diabetes therapies are usually administered according to fixed schedules or patient-adjusted regimens. They do not intrinsically regulate drug release in response to real-time glucose changes [12]. This limitation is evident in subcutaneous insulin therapy, in which standard injections and pump-based systems cannot reproduce the rapid, glucose-dependent insulin secretion of healthy pancreatic β-cells [13].

A primary challenge in insulin therapy is maintaining stable glycemic control despite fluctuating physiological insulin requirements. Insulin needs may vary with nutritional status, physical activity, illness, stress, and individual sensitivity, necessitating ongoing monitoring and dose adjustments to minimize both hyperglycemia and treatment-related hypoglycemia. Moreover, the burden of multiple daily subcutaneous insulin injections may result in poor treatment adherence, compromising effective diabetes management [1,14]. This challenge persists despite the availability of newer microneedle systems, which are more adaptable, acceptable, and user-friendly than traditional methods [15]. Additionally, oral hypoglycemic agents have their own limitations, such as variability in patient response, drug-specific adverse effects, and continued adherence requirements [16].

Given the limitations of conventional diabetes therapies, a shift toward a smart, self-regulated therapeutic system that can automatically respond to real-time changes in blood glucose levels is desirable. Glucose-responsive drug delivery systems represent an innovative approach to diabetes management [12]. They are designed to detect fluctuations in glucose concentration and initiate therapeutic release, thereby increasing drug availability during hyperglycemia while minimizing unnecessary exposure when glucose levels decrease [12,13,17]. Therefore, smart delivery systems have the potential to improve both the precision and safety of diabetes treatment [18].

Within this field, nanomedicine has contributed to significant advancements in the treatment of various medical conditions [19] and is actively being studied in diabetes management [2,20,21]. Glucose-responsive nanomedicine refers to the medical application of engineered nanomaterials to enhance the stability, delivery, and controlled release of therapeutic agents through novel nanocarrier systems [20,22,23,24,25,26]. Glucose-responsive nanomedicine mainly relies on three sensing mechanisms: glucose oxidase, phenylboronic acid, and lectin-mediated mechanisms. Incorporating these mechanisms into nanoscale carriers, including polymeric nanoparticles, nanogels, micelles, liposomes, and hybrid nanostructures, allows these systems to be classified as glucose-responsive nanomedicine [2,12,27,28,29].

This review examines glucose-responsive nanomedicine as an advanced approach to improving diabetes therapy. It focuses on systems that improve glycemic control, reduce unnecessary insulin exposure, and prevent hypoglycemia. The review evaluates key glucose-sensing mechanisms and their use in nanocarriers. It also highlights the strengths and limitations of these platforms, focusing on responsiveness, safety, stability, and their potential for clinical translation.

### Methodology and Literature Search Strategy

This narrative review was informed by a structured literature search conducted using PubMed/MEDLINE, Scopus, and Web of Science. Studies published between January 2000 and 15 July 2026 were considered. The search combined terms related to diabetes and insulin delivery with terms related to glucose-responsive nanomedicine, including “glucose-responsive,” “glucose-sensitive,” “self-regulated insulin delivery,” “nanoparticle,” “nanogel,” “liposome,” “micelle,” “hydrogel,” “microneedle,” “glucose oxidase,” “phenylboronic acid,” and “concanavalin A.” The reference lists and citations of relevant articles were also manually screened to identify additional studies. The final literature search was conducted on 15 July 2026.

English-language, peer-reviewed primary studies and relevant review articles were considered when they addressed glucose-sensing mechanisms, glucose-triggered drug release, nanocarrier design, preclinical evaluation, safety, manufacturing, or clinical translation. Conventional drug-delivery systems without a direct glucose-responsive mechanism, non-diabetes applications, conference abstracts, and non-peer-reviewed publications were excluded from the main analysis. Study selection was based on relevance to the review objectives and the scientific contribution of each article. As this was a narrative review, no formal risk-of-bias assessment or meta-analysis was performed.

## 2. Diabetes Therapy: Current Challenges and Unmet Needs

Despite advances in technology and medicine, maintaining optimal glycemic control remains a major clinical challenge [30]. The worldwide prevalence of diabetes is continually increasing and is associated with significant morbidity and mortality resulting from both microvascular and macrovascular complications [31,32]. Although current therapeutic approaches have improved patient outcomes, many individuals continue to experience inadequate glycemic control, treatment-related adverse effects, and poor patient adherence. Advances in diabetes management, such as improved glycemic control, cardiovascular risk reduction, and earlier detection through screening programs, have contributed to reduced rates of vascular complications. Consequently, life expectancy has increased, contributing to the emergence of additional complications such as cancer, dementia, postoperative infections, and respiratory infections [32].

### 2.1. Limitations of Insulin Injections and Oral Hypoglycemic Drugs

In type 1 diabetes and advanced cases of type 2 diabetes, where oral antidiabetic agents are inadequate for achieving optimal glycemic control, insulin remains the primary and most effective treatment [33]. Insulin therapy replaces deficient endogenous insulin in type 1 diabetes, thereby facilitating glucose uptake into tissues, maintaining serum glucose levels within the target range, and decreasing hepatic glucose production [34]. Although insulin therapy is an integral part of diabetes management, it is predominantly administered subcutaneously; this route of administration is associated with significant limitations, including needle-related anxiety, psychological insulin resistance, and local injection-site complications, all of which compromise treatment adherence and glycemic control, underscoring the need for alternative delivery routes [35]. Studies have shown that when patients with type 2 diabetes are prescribed subcutaneous insulin, they may perceive this as an indication that their condition has “worsened” or that alternative treatments have “failed,” consequently leading to delays in or refusal of treatment. Patient awareness of the potential complications of hypoglycemia, coupled with the requirement for precise insulin administration and comprehensive training, adds further complexity to therapy. This, combined with the demands of frequent glucose monitoring and continuous dose adjustments, contributes to heightened treatment-related anxiety and consequent reluctance to initiate insulin therapy [35].

Although subcutaneous insulin administration is traditionally regarded as the most reliable route, it demonstrates pharmacokinetic variability influenced by external factors. For instance, local blood flow at the injection site can significantly modulate insulin absorption, whereby increased perfusion during heat exposure or after exercise can accelerate systemic absorption, whereas reduced perfusion after exposure to cold temperatures can delay absorption. The injection site can also result in variable absorption rates, as abdominal administration accelerates absorption, whereas thigh and gluteal administration results in delayed absorption due to reduced blood flow in these areas [35].

A common complication of subcutaneous insulin administration is lipodystrophy, a disorder in which repeated injections at the same site result in localized adipose tissue changes [35]. Lipodystrophy falls into two categories: lipohypertrophy, characterized by thickening of the subcutaneous fat, and lipoatrophy, characterized by loss of subcutaneous fat at the injection site. In both cases, localized adipose tissue changes affect insulin pharmacokinetics, specifically influencing the rate of insulin absorption into the bloodstream. This intra-individual variability in insulin absorption causes inconsistent pharmacodynamic responses to the same insulin dose administered at different times, contributing to glycemic excursions and hypoglycemic episodes and resulting in reduced glycemic control [36].

Oral hypoglycemic agents are central to type 2 diabetes management and reduce serum glucose through a variety of mechanisms, including increasing insulin sensitivity in peripheral tissues, stimulating pancreatic β-cells to secrete insulin, increasing urinary glucose excretion, stimulating endogenous incretin release, and reducing glucose and carbohydrate absorption in the gastrointestinal tract [37]. However, the effectiveness of oral hypoglycemic agents is associated with β-cell function, particularly the ability of pancreatic β-cells to produce insulin. Clinical trials such as the UK Prospective Diabetes Study (UKPDS) and A Diabetes Outcome Progression Trial (ADOPT) have demonstrated that the progression of type 2 diabetes mellitus is closely associated with a progressive decline in pancreatic β-cell function. Since the efficacy of oral hypoglycemic agents is somewhat dependent on adequate β-cell activity, the progressive decline in β-cell function results in a subsequent reduction in therapeutic responsiveness, resulting in diminished glycemic control as the disease advances [38].

Oral hypoglycemic agents are associated with class-specific adverse effects that can negatively influence patient adherence and treatment success (Table 1). Biguanides are associated with gastrointestinal side effects and vitamin B12 deficiency [39]. Thiazolidinediones (TZDs) are associated with fluid retention, mediated through increased renal sodium absorption in the collecting ducts, causing peripheral edema and potentially precipitating congestive heart failure. In postmenopausal women, TZDs act through the activation of peroxisome proliferator-activated receptor-γ (PPAR-γ), promoting the differentiation of mesenchymal stem cells into adipocytes and inhibiting osteoblastogenesis, thereby significantly increasing the risk of bone fractures [40]. Sodium-glucose cotransporter 2 (SGLT2) inhibitors reduce renal glucose reabsorption, resulting in glucosuria, which may promote pathogen growth in the urinary tract and increase the risk of genitourinary tract infections. These agents are also associated with acute kidney injury and diabetic ketoacidosis [41].

### 2.2. Poor Glycemic Control and Risk of Hypoglycemia

Poor glycemic control and treatment-induced hypoglycemia arise from the interaction between treatment-related factors and individual patient variability. In T1DM, hypoglycemia arises when standard doses of exogenous insulin are administered despite reduced circulating glucose, such as when meals are delayed or missed, the patient has engaged in prolonged physical activity, or when alcohol is consumed without carbohydrate intake [42]. During exercise, peripheral skeletal muscle glucose uptake increases due to translocation of glucose transporter type 4 (GLUT4) to the cell surface [43]. In the presence of exogenous insulin, this effect is further amplified through insulin-activated GLUT4 activity, subsequently causing a rapid drop in serum glucose levels [44]. The absence of counterregulatory hormonal response, specifically glucagon and catecholamine, prevents the body’s normal physiological mechanism to restore blood glucose during hypoglycemia [45]. During alcohol intake, the liver stimulates ethanol metabolism, subsequently increasing the NADH/NAD^+^ ratio, thereby inhibiting gluconeogenesis, in turn exacerbating the current hypoglycemic state [46].

Oral hypoglycemic agents that are utilized in T2DM also pose the risk of hypoglycemia. Sulfonylureas exert their effects through a glucose-independent mechanism by directly stimulating the release of insulin from pancreatic β cells, irrespective of serum glucose levels. This unregulated insulin release can predispose patients to hypoglycemia [47]. Similarly, Meglitinides bind to sulfonylurea receptor 1 (SUR1) to close ATP-sensitive potassium (K_ATP_) channels, resulting in the secretion of insulin regardless of serum glucose levels [48]. Evidently, conventional standardized therapeutics used in both T1DM and T2DM can cause hypoglycemia [49].

### 2.3. Adherence Issues and Non-Physiological Drug Delivery

**Figure 1:**
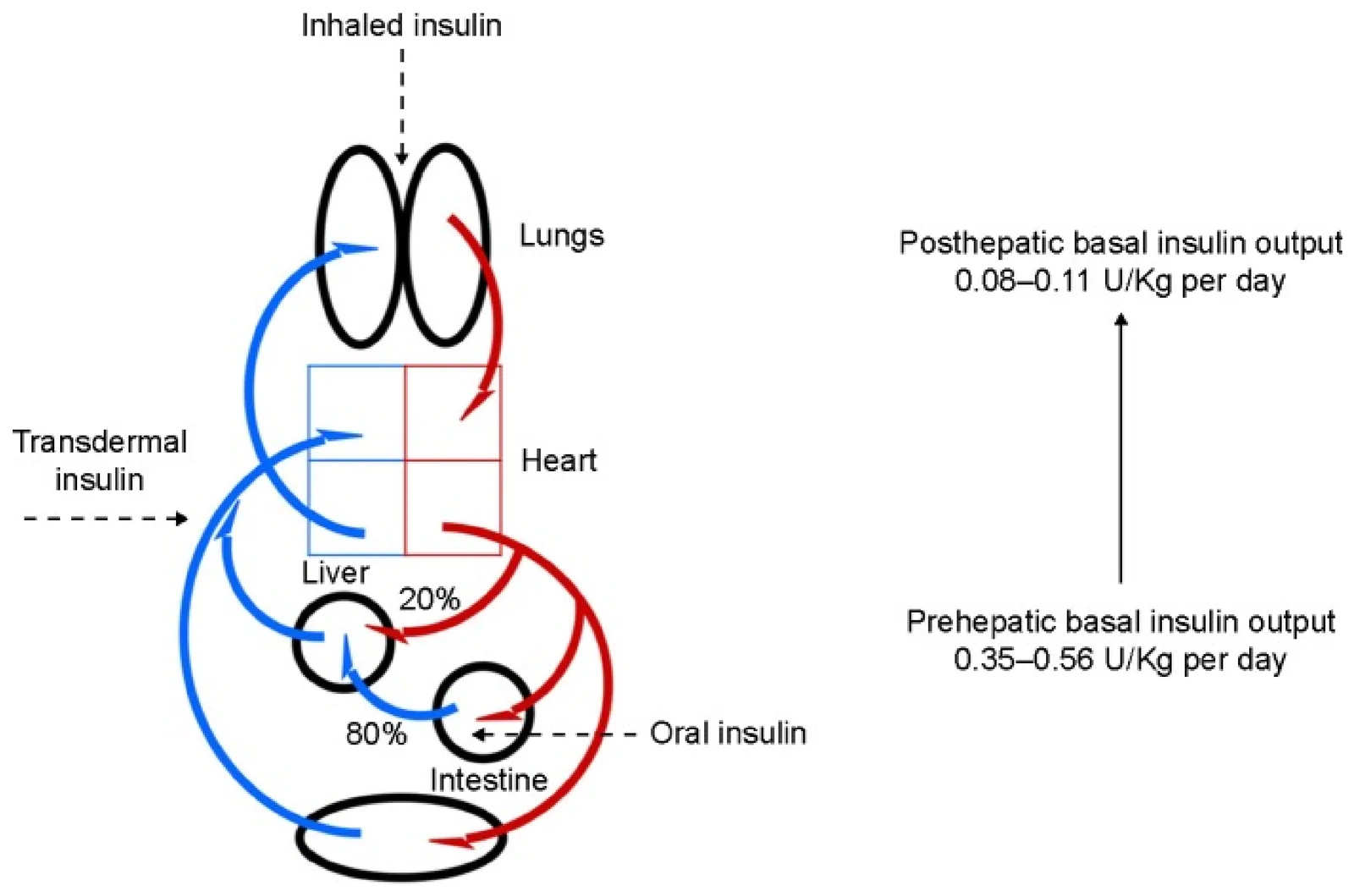
Portal–systemic blood insulin gradient and alternative routes of insulin administration. Red arrows indicate arterial blood flow, blue arrows indicate venous blood flow, and dashed black arrows indicate the inhaled, transdermal, and oral routes of insulin administration. Reprinted from Ref. [51].

Patient adherence issues and the limitations of current diabetic treatments in replicating physiological insulin secretion are major barriers to achieving stable glycemic control [50]. Under normal physiological conditions, insulin is delivered to the liver via the portal circulation, resulting in portal insulin concentrations that are two to fourfold higher than those in the peripheral circulation. Exogenous insulin administration bypasses the portal-hepatic route, resulting in glycemic variability characterized by recurrent episodes of hyperglycemia and hypoglycemia (Figure 1) [51,52].

Accordingly, patients are required to adhere to complex treatment regimens including close blood glucose monitoring, 14, counting, and recurrent dose adjustments (Table 2) [53]. Studies have demonstrated that higher medication regimen complexity was associated with poorer glycemic control and lower patient adherence rates [54,55,56]. Low adherence can manifest in a variety of ways including delayed doses, missed doses, or the intentional reduction in prescribed doses. A survey looking into patient adherence reported that over 50% of T1DM and T2DM intentionally omitted insulin doses, noting that adherence rates decreased as the number of times a day the medication should be taken increased [57]. Consequently, these studies have emphasized the importance of identifying alternative less complex insulin delivery pathways to improve patient adherence.

### 2.4. Need for More Precise and Adaptive Treatment Strategies

**Figure 2:**
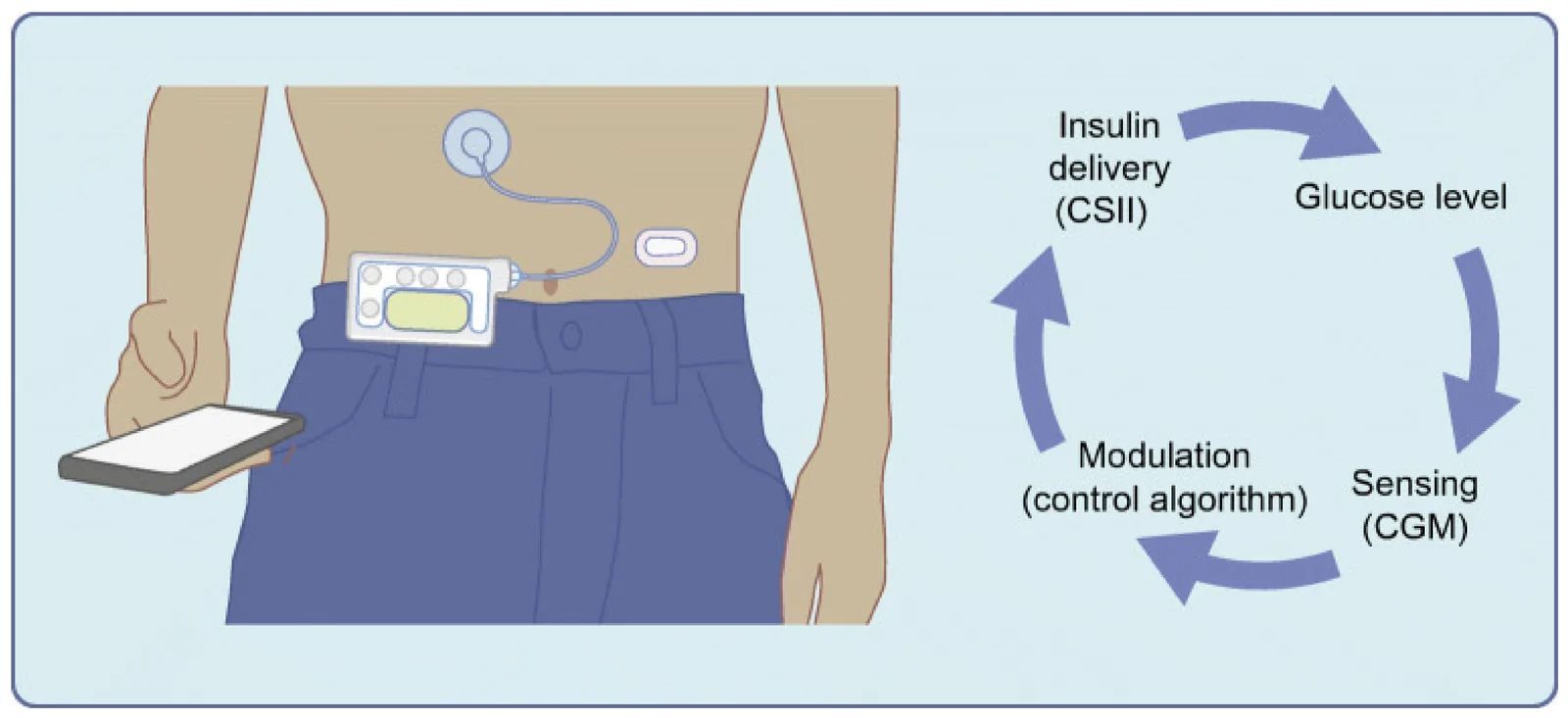
Schematic of the configuration of closed-loop insulin delivery. The circular arrows indicate the direction of the closed-loop feedback process from glucose measurement and sensing to algorithmic modulation and insulin delivery. A continuous glucose monitor (CGM) transmits information about interstitial glucose concentrations to an algorithm hosted on a smartphone or insulin pump that translates information from the glucose sensor and computes the amount of insulin to deliver. An insulin pump delivers a rapid-acting insulin analogue subcutaneously. Insulin delivery is modulated in real time by the control algorithm. Communication between system components is wireless. CSII, continuous subcutaneous insulin infusion. Reprinted from Ref. [59].

Conventional diabetes therapies are associated with several limitations that contribute to clinical challenges, including glycemic variability, treatment-induced hypoglycemia, and suboptimal treatment adherence. Consequently, there is a growing need for individualized and adaptive treatment approaches that can accommodate individual patient characteristics, treatment responses, and changing metabolic demands. Current approaches lack the precision and specificity to account for inter-individual variability in insulin sensitivity and counter-regulatory hormonal responses, resulting in variable glycemic control. Studies have shown the potential of developing a closed-loop artificial pancreas, allowing calculated and individualized delivery of basal insulin and demonstrating evidence of reducing glycemic variability incidences [58]. The closed-loop insulin system utilizes subcutaneous insulin administration; however, it does so according to a calculated algorithm in response to real-time changes in serum glucose levels rather than releasing a standardized amount of insulin with each administration (Figure 2). This system overcomes limitations of conventional therapies including the psychosocial fear of injections and the anxiety associated with traditional subcutaneous administration of insulin. Clinical trials have demonstrated promising outcomes, underscored the potential of personalized medicine, and reinforced the need for continued research and broader clinical implementation in diabetes management [59].

## 3. Principles of Glucose-Responsive Nanomedicine

The development of glucose-responsive nanomedicine has been considered a promising strategy to improve insulin delivery through glucose-dependent drug release. By integrating the glucose sensing and nanocarrier systems, these platforms are designed to achieve more adaptive, efficient, and personalized diabetes management.

### 3.1. Concept and Functional Basis of Glucose-Responsive Nanomedicine

Glucose-responsive nanomedicine (GRN) can be defined as an advanced drug delivery approach designed to release insulin in response to variations in blood glucose concentrations. While conventional methods of insulin delivery rely on scheduled administration, GRN uses an on-demand, feedback-controlled drug delivery process that responds to changes in blood glucose concentrations [60]. The aim of developing GRN-based drug delivery systems is to mimic the function of pancreatic β-cells by releasing insulin according to blood glucose levels [61,62].

For the purposes of this review, a system is considered genuinely glucose-responsive only when glucose directly interacts with a glucose-recognition element and consequently regulates drug release. By contrast, systems that provide only sustained release or respond independently to pH, temperature, degradation, or other stimuli without directly sensing glucose are not classified as glucose-responsive systems [60,61,62,63,64].

**Figure 3:**
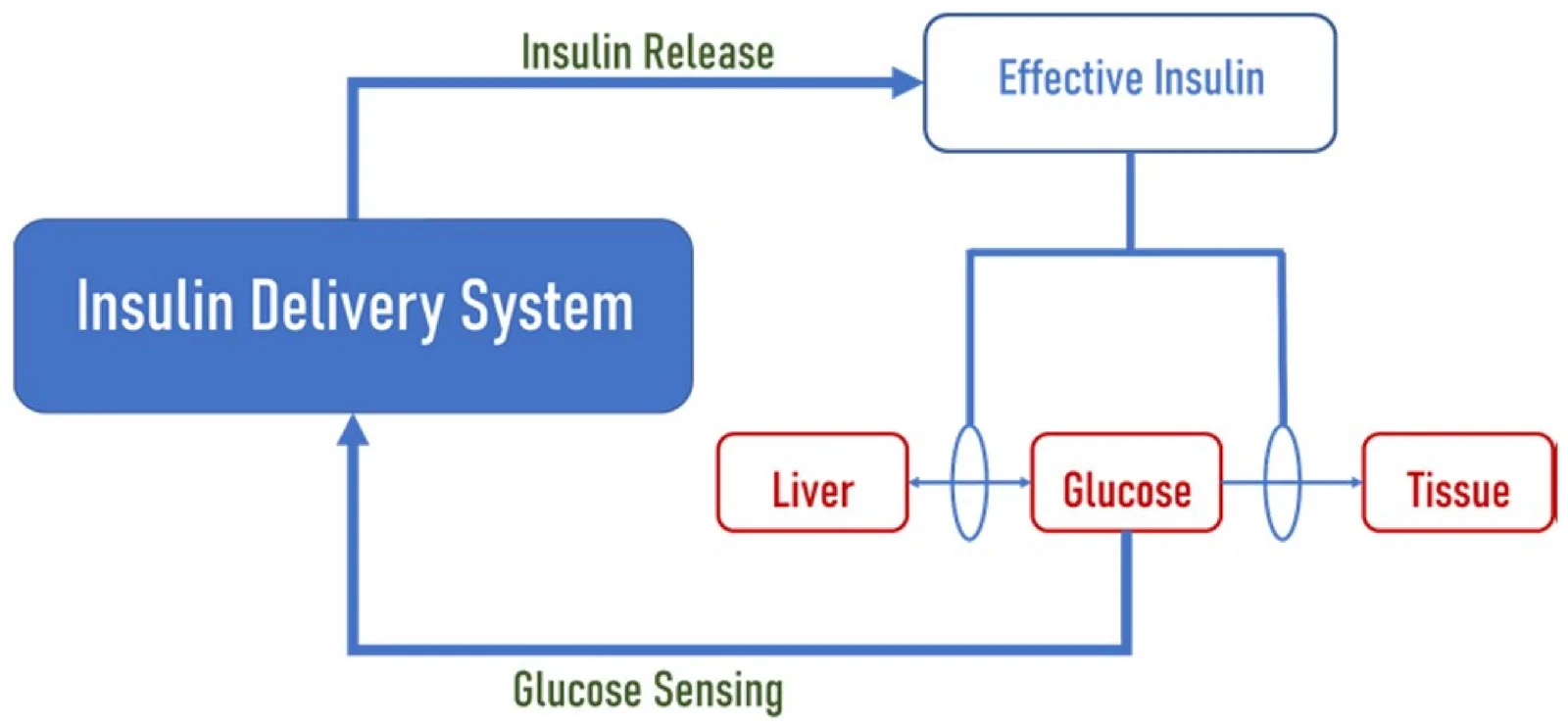
Schematic of a closed-loop insulin delivery system in the body. Blue arrows indicate the direction of the feedback loop: glucose sensing activates the insulin delivery system, which releases insulin, while effective insulin regulates glucose through its effects on the liver and peripheral tissues. Blue boxes represent the insulin delivery system and effective insulin, red boxes identify glucose and the physiological compartments involved in glucose regulation, and green labels identify the glucose-sensing and insulin-release processes. Reprinted from Ref [65].

Typically, β-cells sense blood glucose levels and release insulin when these levels are high. Likewise, glucose-responsive nanocarriers are designed to sense the surrounding glucose environment and release their drug cargo when the glucose level exceeds a certain threshold [63]. The glucose dependence of this process makes GRN-based systems suitable for closed-loop drug delivery, as shown in Figure 3, where drug release can be modulated according to fluctuations in physiological glucose levels without the need for constant external monitoring. In this way, insulin release closely mimics the natural regulatory mechanism of healthy pancreatic tissue [62,64].

### 3.2. How Glucose-Triggered Drug Release Occurs

Glucose-responsive nanomedicine contains three primary functional components: (1) a glucose sensing element, (2) a drug reservoir, and (3) a responsive carrier matrix. The glucose-sensing element detects changes in glucose concentration and translates them into a biochemical signal that induces a structural or physicochemical change in the carrier matrix, subsequently triggering drug release from the reservoir when a predetermined glucose concentration is reached [66,67]. These three components work together to deliver a self-regulating approach for the administration of the desired drug based on demand while minimizing unwanted therapeutic agent exposure [68].

The effectiveness of glucose-responsive nanomedicine relies on its ability to detect changes in glucose levels and produce an appropriate therapeutic response. All these systems share the same general goal: to release therapeutic agents when needed. However, each of those platforms uses a different molecular pathway that interacts with other molecules to be able to interpret changes in glucose concentrations and trigger the release of the appropriate drugs [69]. Several glucose-sensing strategies have been developed to enable this responsiveness, each utilizing distinct biochemical interactions to detect glucose and initiate drug release [70,71].

Currently available glucose-responsive system designs almost always rely on glucose-responsive elements such as phenylboronic acid (PBA), glucose oxidase (GOx), and concanavalin A (ConA). Continued advances in glucose-responsive nanotechnology have increased interest in these systems as potential platforms for future diabetes therapy [61,72].

### 3.3. Benefits over Conventional Delivery Systems

One major advantage of glucose-responsive nanomedicine is its ability to achieve tighter glycemic control as the therapeutic release is responsive to metabolic needs, where glucose concentrations can be maintained within a narrower physiological range [73]. Improved glycemic control is important in the management of diabetes as persistent fluctuations in glucose have been associated with the development of both microvascular and macrovascular complications [74].

Veiseh et al. have pointed out the significance of providing physiologically controlled insulin delivery. Physiological demands for insulin vary during the day depending on the intake of glucose. However, conventional insulin delivery methods have failed to provide accurate physiological delivery. This mismatch may contribute to periods of inadequate glycemic control and excessive insulin exposure. This is addressed by the design of glucose-responsive nanomedicine, which controls the drug release according to the actual glucose level, mimicking the physiological processes of insulin release and glucose control [75].

In addition to better glycemic regulation, glucose-responsive systems can decrease the likelihood of drug-induced hypoglycemia [67]. As glucose levels decrease toward the normal range, less medication is released, thereby reducing unnecessary drug exposure. This mechanism ensures greater safety and becomes one of the major advantages of glucose-responsive devices for delivering insulin [76].

Another important benefit of glucose-responsive nanomedicine is the possibility for better patient compliance and quality of life. Automated drug delivery may reduce the need for frequent therapeutic interventions and improve the management of chronic diseases. Glucose-responsive nanomedicine may also reduce the overall treatment burden and improve the overall quality of the patient experience [64].

### 3.4. Relevance to Precision Diabetes Therapy

Precision medicine focuses on optimizing clinical outcomes by adjusting treatment strategies to meet the physiological requirements of each patient [77]. Controlling blood glucose levels in patients with diabetes remains difficult because glucose levels fluctuate in response to diet, exercise, stress, and disease [78]. This has created a growing need for therapeutic strategies that adjust treatment according to the patient’s physiological state.

GRN is consistent with the goals of precision-based diabetes treatment because it enables dose control according to the patient’s actual blood glucose level rather than a predetermined dosing schedule [79]. This approach may enable personalized diabetes treatment by adapting to changes in metabolic requirements. According to Caturano et al., glucose-responsive nanomedicine represents a promising strategy for precision insulin delivery by enabling treatment to be tailored to real-time physiological conditions [64]. Similarly, Wang et al. stated that glucose-responsive delivery systems are designed to mimic endogenous insulin secretion and improve the accuracy of insulin administration in response to changing glucose levels [68].

The significance of glucose-responsive nanomedicine for precision medicine is further underscored by improvements in smart nanocarrier design. Current-generation nanocarriers can be designed with specific physicochemical properties to improve insulin-delivery efficiency, glucose responsiveness, biocompatibility, and controlled release [73]. Such adaptability enables the development of more personalized therapeutic platforms capable of addressing differences in patient physiology and disease progression, supporting the future transition toward individualized diabetes care [80,81].

## 4. Major Glucose-Sensing Mechanisms

**Figure 4:**
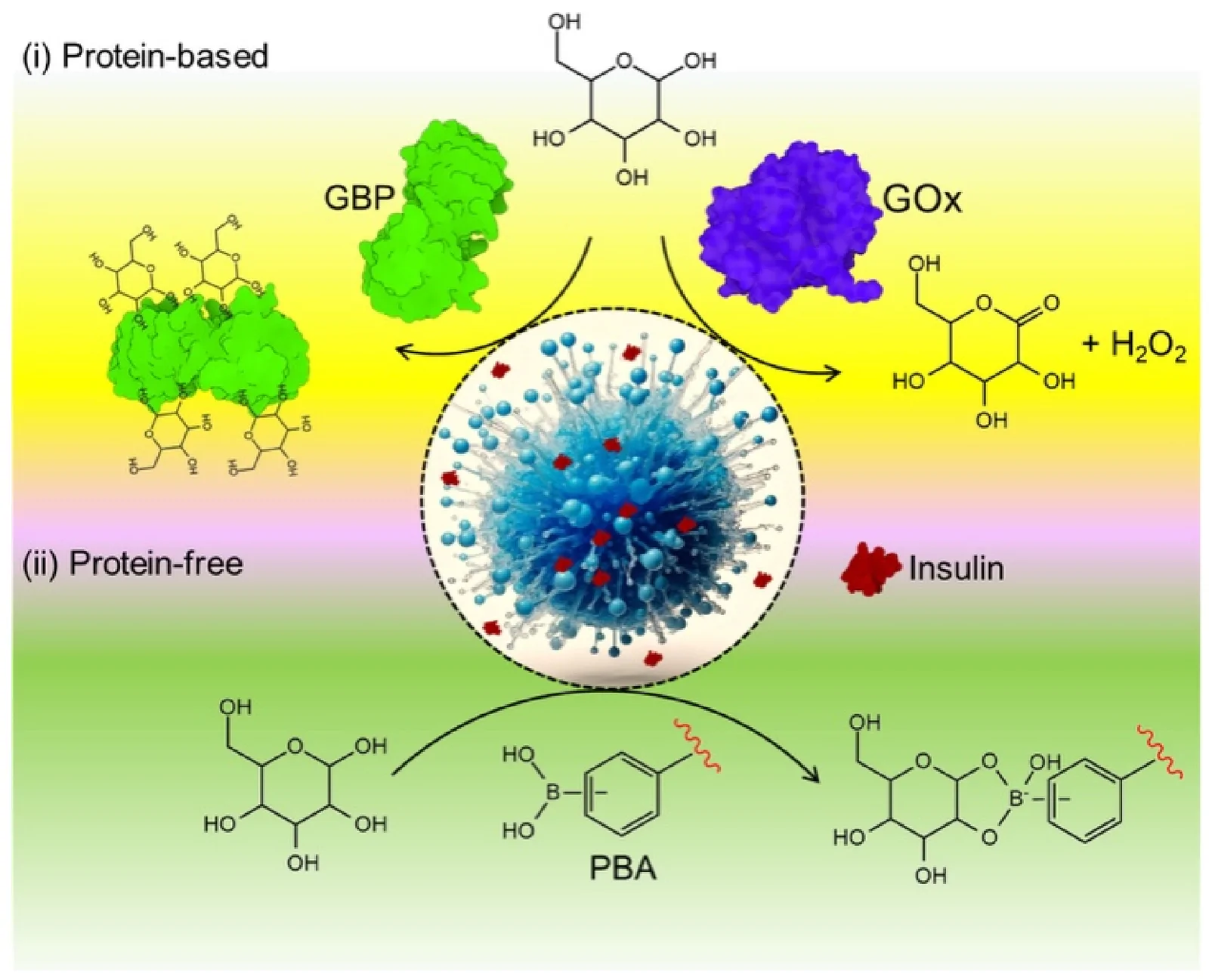
Schematic diagram of glucose-responsive material fabricated from (**i**) protein-based and (**ii**) protein-free strategies. The use of glucose-binding proteins ConA and GOx represents two major examples of protein-based strategies. The second design uses PBA-based molecular receptors to impart glucose-responsive properties. Reprinted from Ref. [82].

The glucose-sensing mechanism is the core of any glucose-responsive nanocarrier, and it determines how the system detects rising glucose levels and translates that signal into active insulin release. There are three main mechanisms by which these systems operate: glucose oxidase (GOx)-based systems, phenylboronic acid (PBA)-based systems, and concanavalin A (ConA)-based systems. Each of these groups differs in terms of sensitivity, stability, physiological compatibility, and immunogenicity risk. The mechanism chosen determines the entire downstream system, from sensing to what kind of nanocarrier can be used, the path of administration (injected, oral, skin patch), and the rules related to drug storage. In addition to these subtopics, there are emerging systems and hybrid mechanisms that have been developed to address the limitations of individual mechanisms. Figure 4 illustrates the fundamental distinction between protein-based strategies, which use GOx or ConA, and protein-free strategies, which rely on PBA. As shown, protein-based strategies incorporate the sensing element as an active biochemical component within the carrier, whereas protein-free PBA-based strategies use small synthetic molecules, reducing the risk of immunogenicity but requiring precise chemical engineering [82,83,84,85].

### 4.1. Glucose Oxidase (GOx)-Based Systems

**Figure 5:**
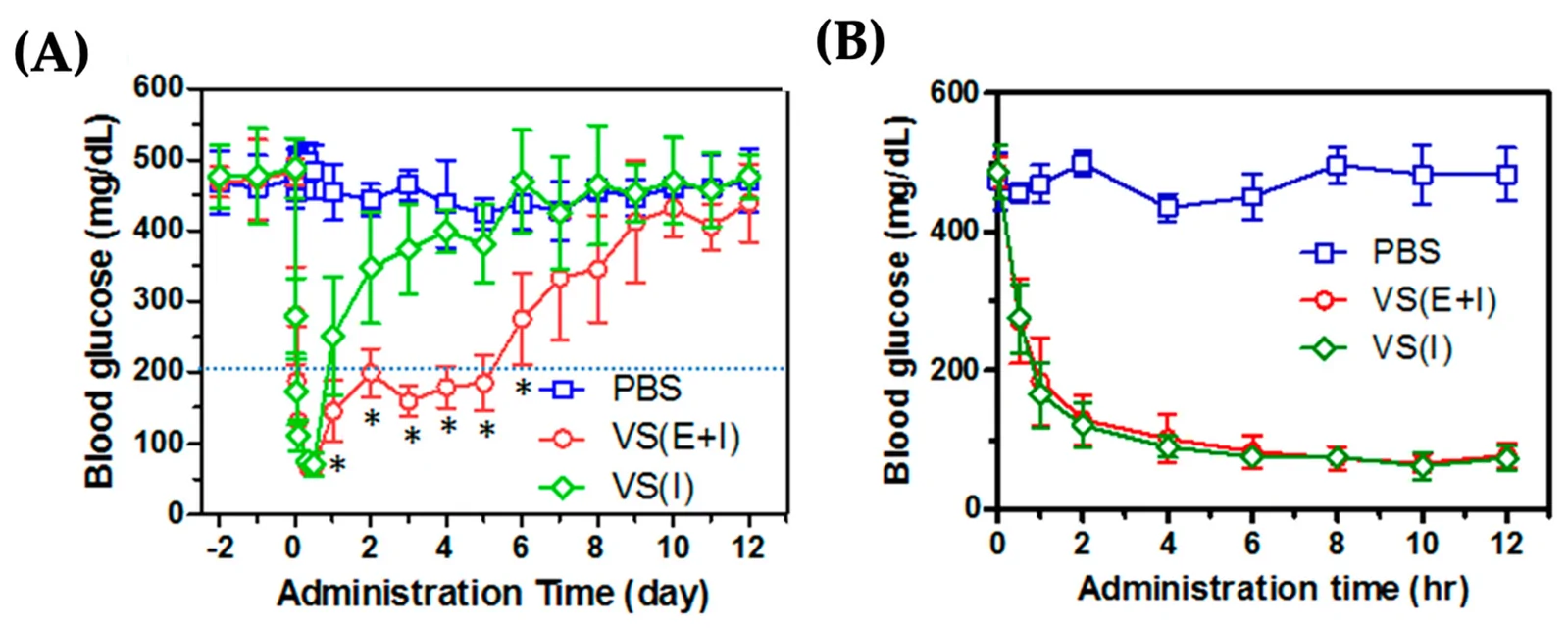
In vivo glucose regulation by nanovesicles in STZ-induced diabetic mice. (**A**) BG levels in STZ-induced diabetic mice after treatment with PBS solution, vesicles encapsulating both enzyme and insulin [VS(E+I)], or vesicles encapsulating insulin only [VS(I)]. *p* < 0.01 compared with the PBS group. (**B**) BG levels were continuously monitored during the first 12 h after administration of PBS solution, VS(E+I), or VS(I). Data represent the mean ± SD (n = 7). Abbreviations: BG, blood glucose; PBS, phosphate-buffered saline; SD, standard deviation; STZ, streptozotocin; VS(E+I), vesicles encapsulating enzyme and insulin; VS(I), vesicles encapsulating insulin only. Reprinted from Ref. [89]. An asterisk (*) indicates *p* < 0.01 compared with the PBS group.

GOx is a flavoprotein enzyme that is derived from the fungus *Aspergillus niger*, with the role of oxidizing glucose. The mechanism of this system is the conversion of glucose into glucono-δ-lactone which rapidly breaks down into gluconic acid. This product is responsible for a pH drop locally, from ~7.4 to ~6.5. H_2_O_2_ is also formed as a byproduct of this reaction. These two products then act as separate triggers: the pH drop causes pH-sensitive polymers in the carrier to swell and open, while the H_2_O_2_ cleaves oxidation-sensitive chemical bonds, also opening the carrier. Catalase is frequently co-encapsulated to decompose H_2_O_2_ into water and oxygen, which may reduce oxidative damage and partially replenish oxygen. However, catalase does not eliminate the risks associated with transient H_2_O_2_ accumulation, and regenerated oxygen may also weaken hypoxia-dependent release mechanisms. System performance therefore depends on oxygen availability, enzyme stability, diffusion, and the relative concentrations of GOx and catalase. To maintain enzymatic activity in vivo, GOx must be immobilized within the carrier architecture rather than freely loaded. To do this, strategies include covalent grafting to the polymer matrix and encapsulation within a protective polymeric shell [83,84,85,86,87]. One glucose molecule generates both an acid and an oxidant, which is considered a strong and amplified signal from a small input. This is why this system is commonly used in injectable nanoparticles and transdermal microneedle patches, where the local pH shift stays contained within the carrier [84,87,88]. Tai et al. loaded GOx, catalase, and insulin into pH-sensitive polymer nanovesicles, where GOx converts glucose to gluconic acid, dropping local pH and triggering insulin release through disassembly. Results showed that a single subcutaneous injection was able to maintain normal blood glucose levels in diabetic mice for up to 5 days with no inflammatory response, as shown in Figure 5 [89]. This study demonstrated that a purely enzymatic pH-driven mechanism is sufficient to achieve sustained glycemic control without needing synthetic chemical triggers. Table 3 summarizes the major GOx-based carrier platforms and their corresponding glucose-triggered release mechanisms [85,90].

Despite their signal-amplifying capacity, GOx-based systems face several physiological limitations. Restricted oxygen availability may reduce glucose oxidation, while excessive H_2_O_2_ can damage surrounding tissues and decrease enzyme activity. Enzyme denaturation, leakage and immune recognition may further reduce long-term stability. Moreover, diffusion of glucose and oxygen through the carrier and surrounding tissue can delay insulin release compared with responses measured under simplified in vitro conditions [86,87,90].

### 4.2. Phenylboronic Acid-Based Systems (PBA)

**Figure 6:**
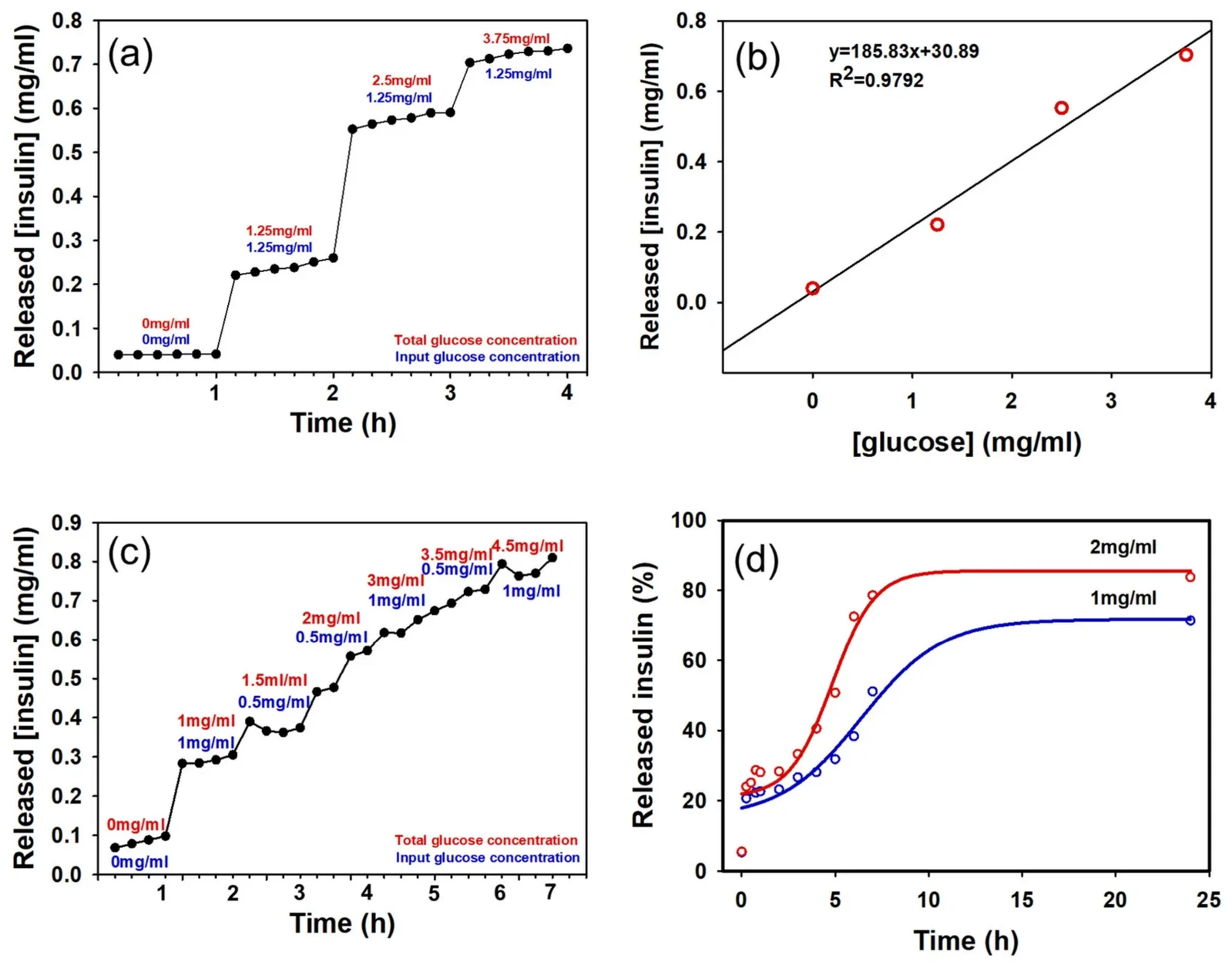
In vitro glucose-responsive release of insulin from POSS-APBA@Insulin NPs. (**a**,**c**) Continual insulin release; (**b**) typical insulin-release curves at glucose concentrations of 0–3.75 mg/mL; and (**d**) insulin release at glucose concentrations of 1 and 2 mg/mL at pH 7.4 and 37 °C. Abbreviations: APBA, 3-aminophenylboronic acid; NPs, nanoparticles; POSS, polyhedral oligomeric silsesquioxane. Reprinted from Ref. [94].

PBA is a small synthetic molecule containing a boronic acid group attached to a phenyl ring. This forms reversible covalent bonds when encountering glucose, specifically with the two adjacent hydroxyl groups on the glucose molecule. In contrast to GOx, PBA contains no protein component, which allows for more chemical stability and less immunological reaction risk. In terms of mechanism, PBA forms cross-links with the carrier’s polymer network, keeping it compact and sealed under normal glucose levels [91,92]. When glucose rises, free molecules compete for the PBA binding sites, breaking the cross-links, increasing the network’s hydrophilicity and causing osmotic swelling and carrier disassembly, releasing insulin. The main engineering limitation of this structure is that native PBA only reaches its glucose-binding tetrahedral form above its pKa (~8.8), which is too alkaline for the human body [85,93]. Strategies to improve glucose binding at physiological pH include introducing electron-withdrawing substituents that lower the pKa of the boronic acid group, incorporating neighboring amines that stabilize boronate formation, and using multivalent PBA architectures. However, these modifications do not necessarily guarantee sufficiently selective or rapid glucose responsiveness under physiological conditions [91,92,93,94]. This is preferred when a fully synthetic and stable system is required. It is incorporated across many carrier types like nanoparticles, micelles, nanogels, and transdermal hydrogel patches [83]. Table 4 summarizes PBA derivatives and their corresponding hydrogel platforms over the past ten years, highlighting combinations used to achieve glucose-responsive drug delivery over the past decade [95]. Kim et al. developed POSS-APBA nanoparticles that self-assemble into micelles at pH 7.4 (physiological pH), achieving 73.2% insulin entrapment efficiency. Insulin release was found to be directly proportional to glucose concentration across the clinically relevant range, confirming that substituent-driven pKa shifting of the boronic acid group alone is sufficient for precise glucose-responsive delivery without any enzymatic component (Figure 6) [85,94].

PBA derivatives recognize cis-diol structures rather than glucose exclusively. Consequently, competing sugars and glycosylated biomolecules in biological fluids may occupy PBA-binding sites and reduce glucose selectivity. Binding and release are also affected by local pH, ionic strength, protein adsorption and the accessibility of PBA groups within the carrier. Therefore, glucose responsiveness demonstrated in simple buffer solutions may overestimate performance under physiological conditions, and selectivity, basal leakage and repeated reversibility should be evaluated in biologically relevant media [92,93,94].

### 4.3. Lectin/Concanavalin A-Based Systems (ConA)

**Figure 7:**
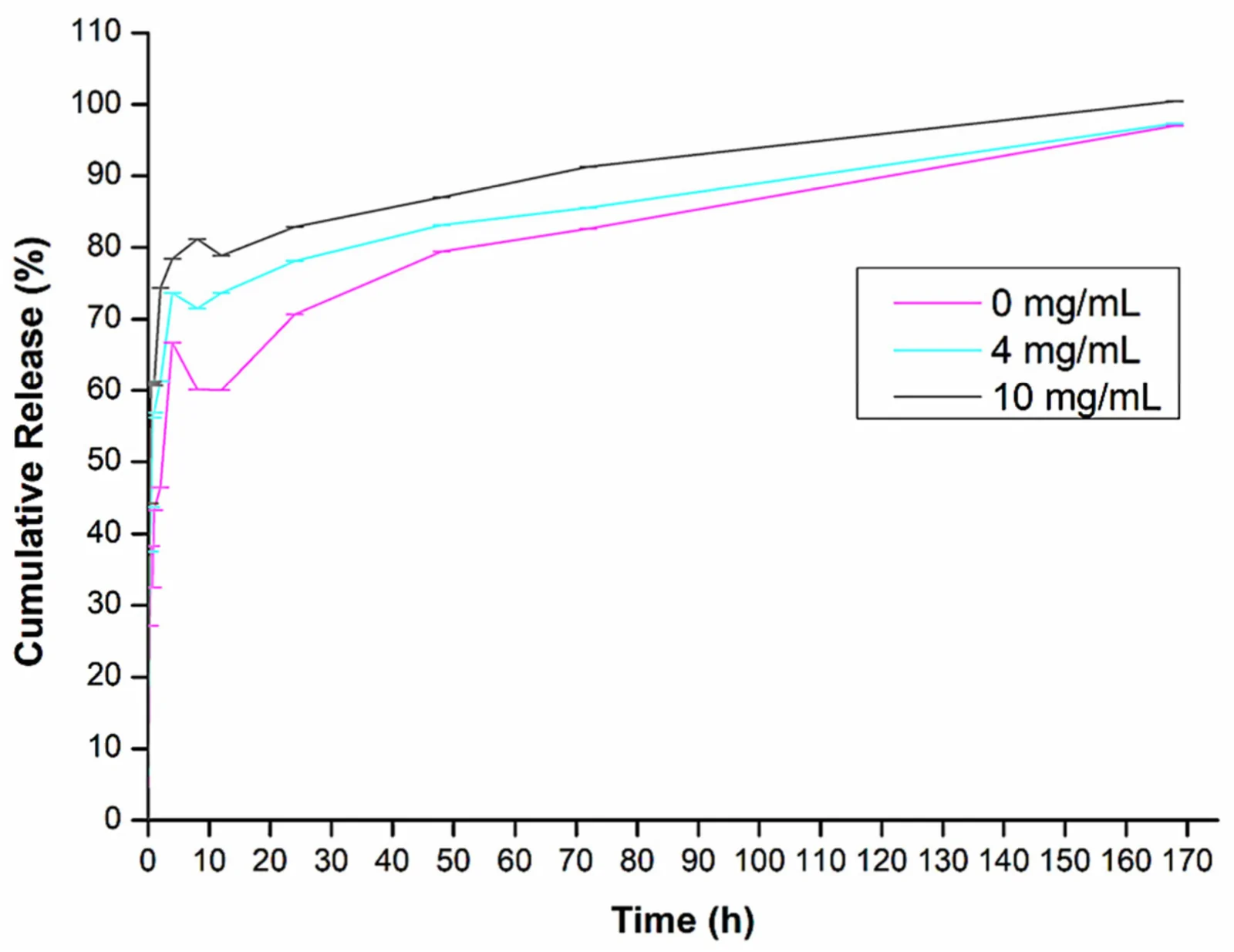
In vitro insulin release profiles from the hydrogel in solutions of differing glucose release media concentrations at 37 °C. Reprinted from Ref. [101].

Lectins are proteins that bind carbohydrates; Concanavalin A (ConA) is the most studied of the lectin family. ConA is extracted from jack beans, and exists in tetramer form, with each subunit containing a metal-ion-dependent glucose-binding site that works in coordination with examples of metal ions like Ca^2+^ and Mn^2+^ [96,97]. Although ConA can bind glucose at physiological pH, its affinity is not exclusive to glucose and depends on pH, ionic conditions and divalent metal ions [96,97,98]. ConA is cross-linked with glycosylated insulin or a glycopolymer to form a stable network that can hold insulin in place under normal glucose levels. When glucose levels rise, free glucose molecules competitively displace the glycopolymer from ConA, weakening the network in a manner that is concentration-dependent, as competitive binding equilibrium shifts with rising glucose, releasing insulin proportionally [84,99]. This system is primarily used within injectable hydrogel depots and implantable reservoir devices, where spatial confinement of the gel matrix ensures that competitive displacement produces a controlled release rather than passive diffusion [100]. Mansoor et al. conducted an experiment where ConA was loaded into a chitosan–Pluronic F-127 hydrogel, demonstrating glucose-proportional insulin release across simulated glycemic conditions over one week, reaching 97% cumulative release at hyperglycemic levels with >80% cell viability in pancreatic cell lines (Figure 7). However, ConA leakage, non-specific carbohydrate binding, structural instability, immunogenicity and mitogenic T-cell activation remain important barriers to clinical translation [100,101].

### 4.4. Hybrid and Emerging Glucose-Responsive Mechanisms

Each main mechanism mentioned has a specific limitation. For example, GOx uses oxygen, PBA has slower binding kinetics compared to enzymatic systems, and ConA can cause inflammation [101]. Hybrid systems are emerging as the new phase of glucose-responsive mechanisms, as they combine two mechanisms to mitigate their individual limitations. The goal of this field is faster, more precise release with fewer side effects [85]. Table 5 maps out representative dual-responsive injectable hydrogel systems that combine glucose-sensing with secondary stimuli, demonstrating expanded release duration and improved glycemic control relative to single-mechanism systems. More recently, research has moved toward nanoparticle-based hybrid designs, where the smaller scale and tighter structural control of these carriers allow for a faster and more tunable release response compared to hydrogel platforms [82,83].

GOx and Hypoxia: These systems exploit the fact that GOx consumes oxygen, and this depletion acts as a second trigger, causing reductive cleavage of hypoxia-sensitive linkers in the polymer, accelerating the release of insulin. This mechanism is faster than GOx alone [84,102,103].

Dual pH and H_2_O_2_ Responsiveness: These systems are designed to respond simultaneously to both byproducts of the GOx reaction rather than relying on a single trigger. Each trigger acts on a distinct structural component, with pH affecting ionizable polymer groups and H_2_O_2_ acting on oxidation-sensitive linkages. Neither trigger alone fully dismantles the carrier, but together they promote near-complete carrier disassembly. This combined response may reduce unwanted drug leakage at normal glucose levels [71,84,88,104,105].

PBA and Supramolecular Assembly: These systems combine the glucose-binding ability of PBA with host–guest chemistry, in which molecules self-assemble into a stable structure that dissociates when glucose levels rise. A cyclodextrin ring accommodates a PBA-tagged component through host–guest interactions, forming a stable nanoparticle at normal glucose levels. When glucose concentrations increase, glucose binding disrupts these interactions, destabilizing the assembly and triggering insulin release [105,106,107,108,109].

PBA-Insulin Conjugates: Rather than encapsulating insulin within a carrier, this approach modifies insulin by attaching PBA directly to the insulin molecule. At normal glucose levels, PBA promotes the binding of insulin to albumin in the blood, limiting its availability. Rising glucose concentrations alter this interaction and promote the release of insulin into circulation [110,111]. For broader clinical context, MK-2640 (Merck), which is an insulin-saccharide conjugate rather than a PBA-insulin conjugate, reached Phase 1 trials but did not demonstrate sufficient glucose-responsive performance for clinical use. This outcome illustrates the broader challenge of tuning glucose-binding affinity to the euglycemic range (~4–10 mmol/L) while maintaining reversible binding [84,112].

The three glucose-sensing mechanisms examined in this section are GOx, PBA, and ConA. Each has a distinct trade-off profile in terms of response speed, physiological compatibility, and immunogenic risk. None of these individual mechanisms alone meets all the requirements of an ideal clinical system. GOx produces a strong biochemical signal but raises concerns regarding enzyme stability and immune responses in vivo. PBA avoids these enzyme-related limitations but often requires structural modification to function effectively at physiological pH. ConA provides natural glucose-binding capability but has not advanced clinically because of its mitogenicity and associated safety concerns. Hybrid and emerging systems have therefore been developed to address these limitations by combining multiple sensing elements, aiming to achieve broader operating ranges, more appropriate response thresholds, and improved safety. The nanocarrier cannot be designed independently of the sensing mechanism because its composition, assembly, and drug-release behavior are constrained by the mechanism selected from the outset [82,83,84,85].

Although GOx-, PBA-, and ConA-based systems all enable glucose-responsive drug delivery, they differ considerably in their glucose-sensing mechanisms, physicochemical characteristics, and translational potential. To facilitate direct comparison, Table 6 summarizes the principal strengths, translational challenges, and clinical implications of the major glucose-sensing mechanisms discussed in this review.

Each method for glucose detection has its advantages and disadvantages that define its utility for clinical use. The GOx-based systems show excellent glucose sensitivity but are limited by enzyme-related issues such as oxygen dependence and hydrogen peroxide production. PBA-based systems are a promising and protein-free alternative with improved design flexibility but need further optimization to enable effective glucose sensing in physiological conditions. ConA-based systems have good glucose-binding properties but are mainly limited by immunogenicity and biocompatibility problems. Thus, the current research is increasingly orientated towards hybrid platforms that integrate complementary sensing mechanisms to achieve better responsiveness, alleviating the constraints of individual systems. Because the sensing mechanism determines the material requirements and release behavior of the delivery system, the following section examines the nanocarrier architectures used to implement these glucose-responsive mechanisms.

## 5. Nanoparticles and Platform Design

Various nanoparticle platforms have been studied for drug delivery. Each formulation of the platform provides a unique characteristic in terms of drug encapsulation and loading, stimuli-responsiveness and release performance. Examples of nanocarrier platforms include: (i) polymeric nanoparticles, (ii) nanogels, (iii) liposomes, (iv) micelles, and finally (v) multifunctional platforms.

### 5.1. Polymeric Nanoparticles

Polymeric nanoparticles are made from biodegradable polymers, including chitosan, alginate, poly(lactic-co-glycolic acid) (PLGA), dextran, and hyaluronic acid. These particles generally range from 1 to 100 nm in size, although particles up to approximately 200 nm are commonly used in biomedical applications. They are bioengineered to improve drug protection and enhance targeted bioavailability depending on varying body conditions. In terms of drug loading, the therapeutic agent can either be entrapped in the polymeric matrix (the wall of the nanoparticle) or embedded in the core by surface adsorption and chemical attachment to the polymer surface. The drug is then released in specific ways, including the diffusion of the drug out of the encapsulation, the degradation of the polymer, and the swelling of the polymer [113].

**Figure 8:**
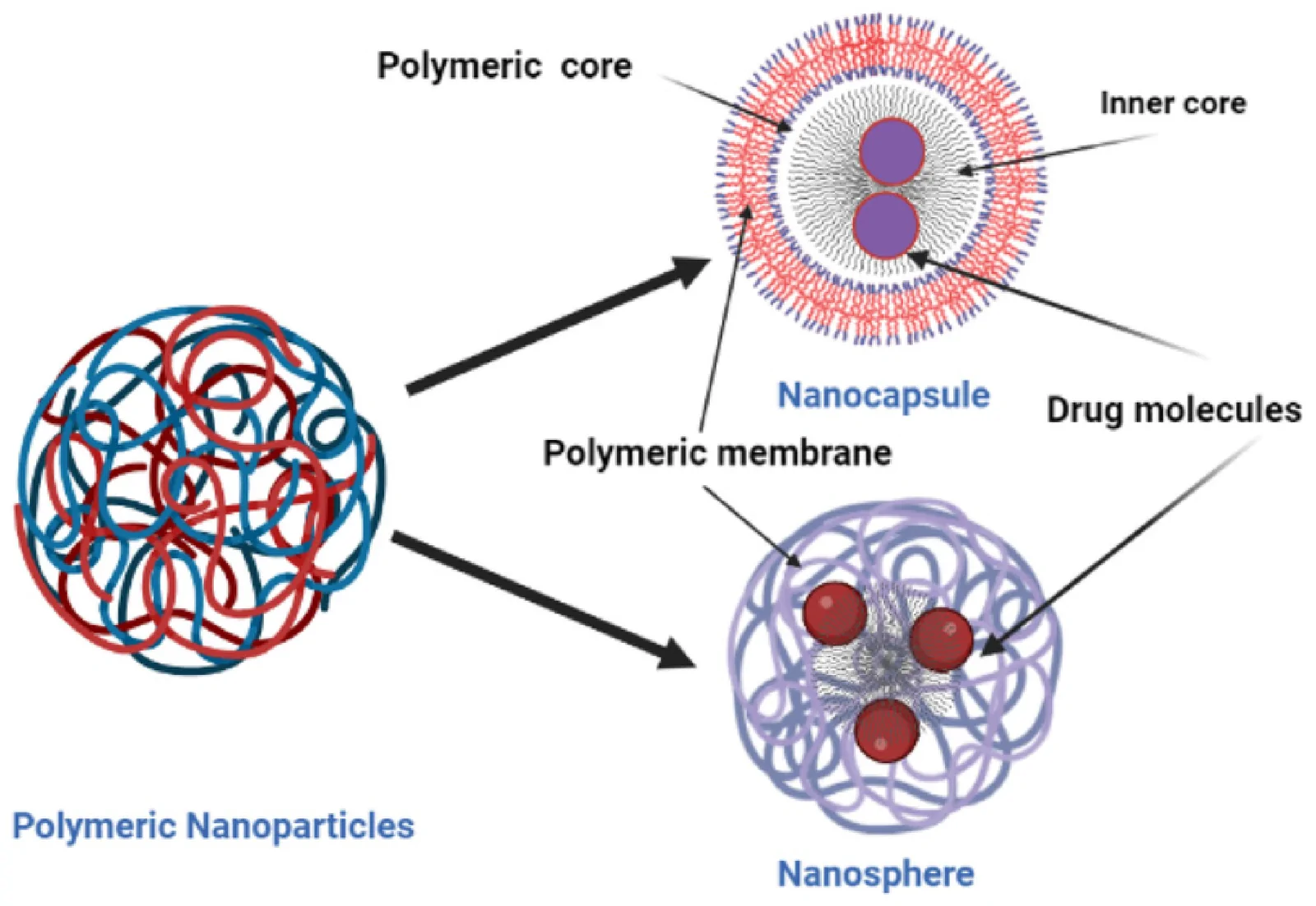
Schematic representation of the structure of nano-capsules and nanospheres. Reprinted from Ref. [114].

Polymeric nanoparticles are generally classified into nanocapsules and nanospheres. Nanocapsules are core–shell structures consisting of a polymeric membrane surrounding a core that contains the therapeutic agent. In contrast, nanospheres consist of a polymeric matrix in which the drug is uniformly dispersed throughout the polymeric network rather than concentrated within a central core (Figure 8). These structural differences influence the drug-loading mechanism and release profile [114].

**Figure 9:**
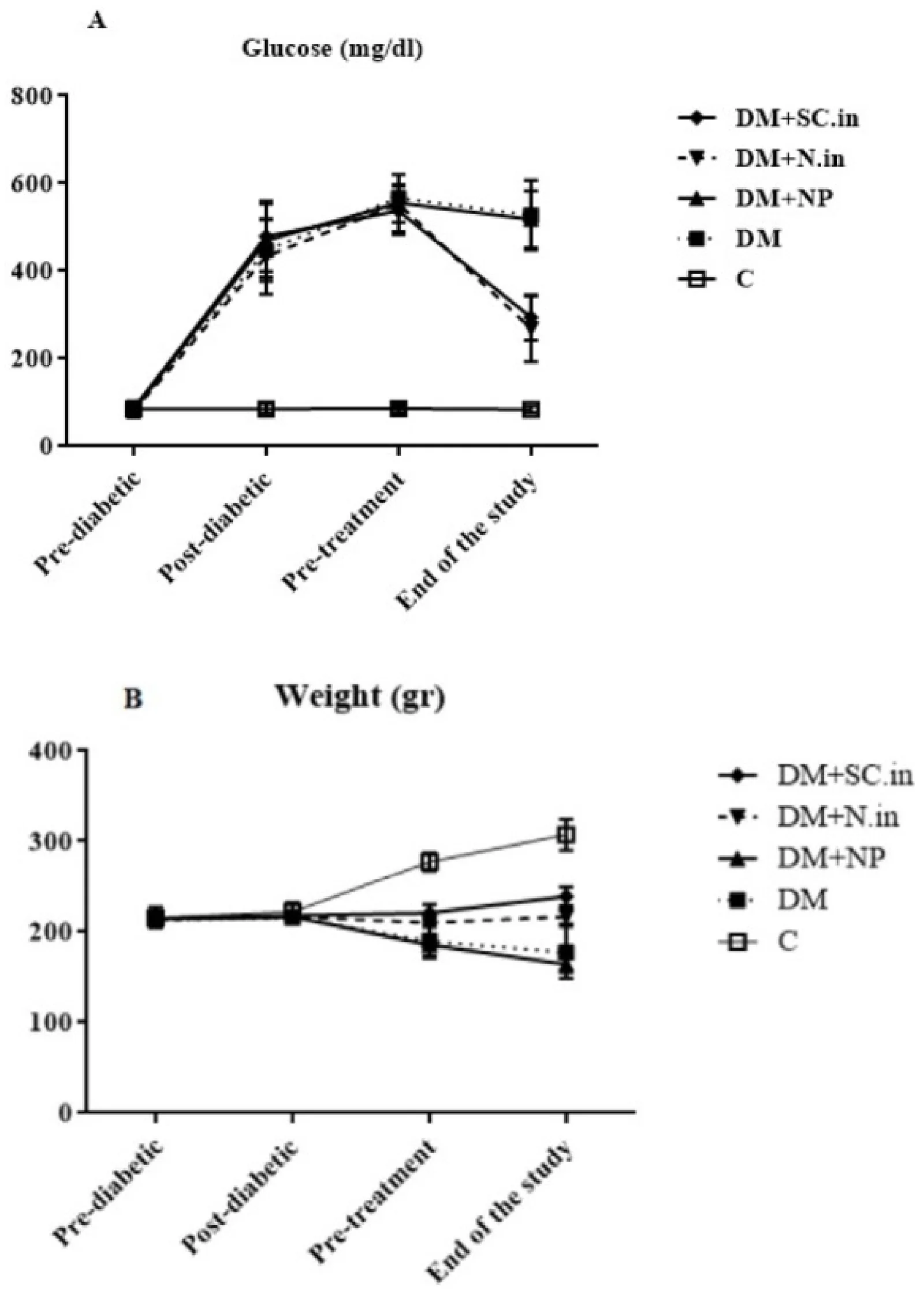
(**A**) shows glucose levels and (**B**) shows weight changes during 10 weeks in all groups. Abbreviations: C = Normal control, DM = Diabetic control, DM+NP = Diabetic group treated with nanoparticle, DM+N.in = Diabetic group treated with oral nanoinsulin, DM+SC.in = Diabetic group treated with subcutaneous injected insulin. Reprinted from [115].

Polymeric nanoparticles have been used to deliver various drugs including anti-cancer and antibiotics. In diabetes research [29]. Heidarisasan et al. performed an in vivo experiment on diabetic rats, evaluating insulin-loaded trimethyl chitosan nanoparticles (polymeric nanoparticles), and found that they improved both renal and oxidative stress markers when compared to conventional injectable insulin. Additionally, results show that there has been greater control over hyperglycemia in nanoparticle formulations, even though the difference was not statistically significant (Figure 9) [115].

### 5.2. Nanogels

Nanogels are defined as soft, nanosized hydrogel particles that consist of crosslinked polymer networks holding a high-water content. This unique composition provides excellent biocompatibility, making them highly effective for encapsulating hydrophilic therapeutics like insulin and various biomacromolecules. Their three-dimensional hydrated structure is capable of holding medications while maintaining their biological activity, and through specific structural adjustments, they can even be adapted to carry hydrophobic agents. A primary benefit of nanogels is their stimulus-responsive behavior. Drug release from these particles can be activated via multiple pathways including diffusion, the breakdown or degradation of the polymer network, pH-induced swelling or shrinking, ion displacement, or the application of external energy input. Each of these triggers modifies the gel structure to facilitate the release of the loaded drug [116,117,118].

Within the field of diabetes therapy, this responsive nature is highly beneficial. Nanogels can be specifically designed to initiate insulin release when exposed to glucose-related triggers, such as fluctuations in glucose concentration, shifts in local pH, or specific enzyme-mediated reactions. Consequently, nanogels have emerged as promising self-regulated platforms designed for controlled antidiabetic drug delivery [119].

Li et al. developed glucose and hydrogen peroxide-responsive nanogels for insulin delivery. In this system, glucose oxidase converts glucose into gluconic acid and hydrogen peroxide, and the generated hydrogen peroxide destabilizes the oxidation-sensitive nanogel, thereby accelerating insulin release. The nanogels demonstrated glucose-dependent insulin release, favorable biocompatibility, and glucose-lowering activity following subcutaneous administration in diabetic mice [120]. This system therefore meets the definition of genuine glucose responsiveness because insulin release is regulated by glucose recognition and its downstream biochemical products.

### 5.3. Liposomes

**Figure 10:**
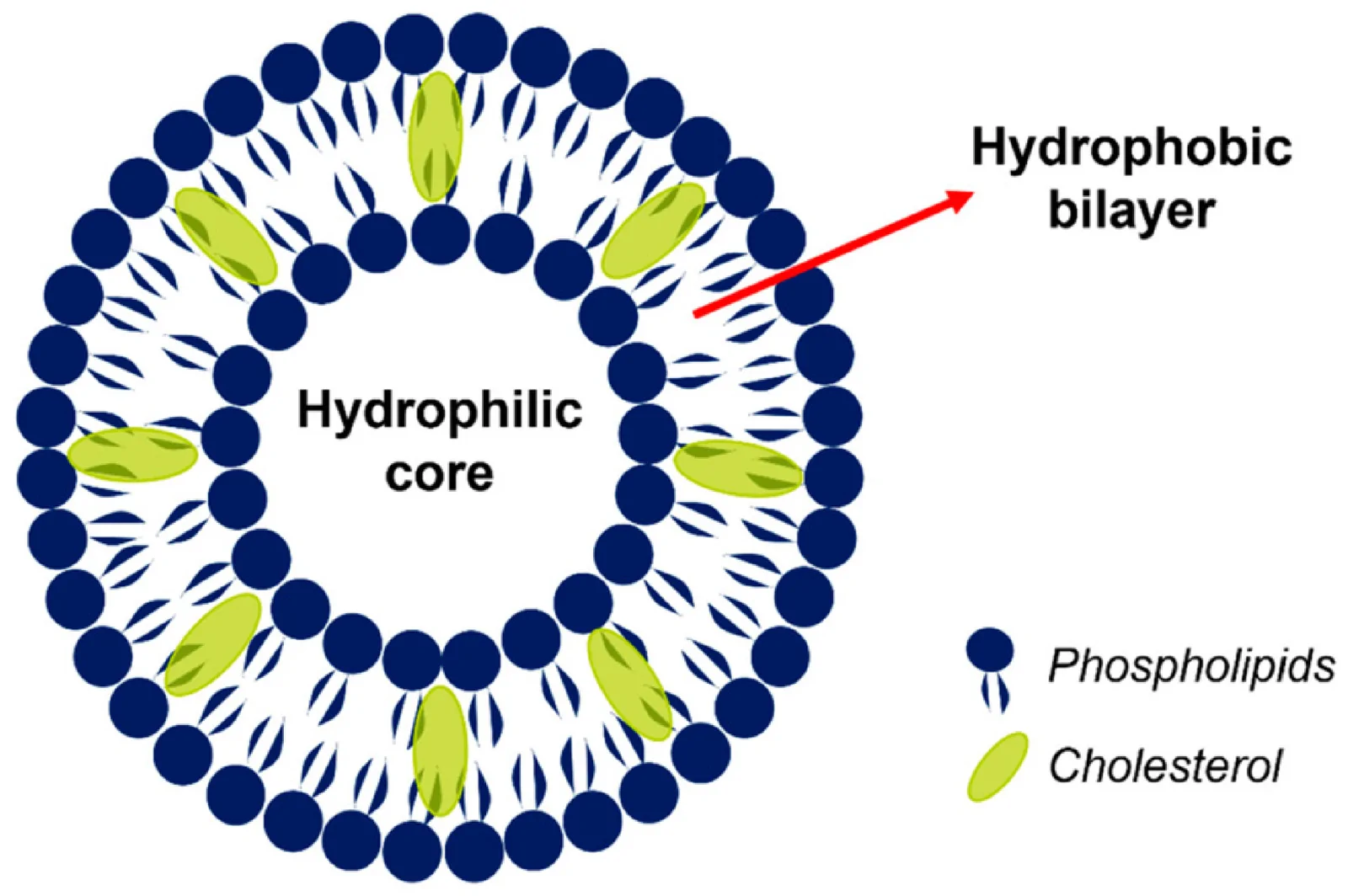
Schematic structure of a liposome. The hydrophilic core is enclosed by a hydrophobic bilayer made of phospholipids and cholesterol, enabling the delivery of both hydrophilic agents within the core and hydrophobic agents within the bilayer. Reprinted from Ref. [121].

Liposomes are nanosized vesicular carriers made up of one or more phospholipid bilayers that surround an aqueous core. Their structure mimics biological systems, they demonstrate excellent biocompatibility, and they possess the capacity to hold both hydrophilic and hydrophobic drugs, making them attractive options for drug-delivery systems. Hydrophilic drugs are typically inserted into the aqueous core, whereas hydrophobic drugs are embedded within the lipid bilayer. Figure 10 demonstrates the structure of a liposome made from a lipid bilayer [121]. Following administration, drug release can occur through several pathways including diffusion, destabilization of the membrane, fusion with biological membranes, or breakdown of the liposomal structure [122].

In the context of diabetes therapy, liposomes have garnered attention as carriers for insulin and other antidiabetic medications. Their structure can be adjusted to produce stimulus-responsive systems, which enable drug release when exposed to glucose-related triggers such as variations in glucose concentration, pH changes, or enzymatic reactions. This characteristic establishes liposomes as valuable platforms for controlled and potentially self-regulated antidiabetic drug delivery [123].

Liu et al. created glucose-responsive multivesicular liposomes intended for insulin delivery and demonstrated encouraging results in both laboratory and animal studies. In laboratory experiments, the system released increased amounts of insulin at elevated glucose concentrations, exhibited pulsatile release when glucose levels changed, and remained fairly stable at physiological pH. In animal experiments, using rats with chemically induced type 1 diabetes, the insulin-loaded liposomes swiftly decreased blood glucose, sustained it within the normal range, and showed superior performance compared to free insulin in glucose tolerance tests, with glucose returning to normal in roughly 2 h following the challenge. These observations demonstrate that glucose-responsive liposomes serve as a valuable intelligent platform for insulin delivery in diabetes [124].

### 5.4. Micelles

Micelles are self-assembled nanosized carriers formed from amphiphilic molecules that possess a hydrophobic interior and a hydrophilic exterior. They are particularly well-suited for drugs with limited water solubility because the hydrophobic interior can hold lipophilic compounds, while the outer layer enhances stability and biocompatibility. Beyond improving solubility and bioavailability, micelles can shield the enclosed drug and facilitate controlled release. Drug release typically occurs through diffusion, breakdown of the micelle structure, degradation of the polymer, or changes triggered by external stimuli [125].

Gaballa and Theato developed glucose-responsive polymeric micelles assembled through reversible boronic acid–diol interactions between a phenylboronic acid-containing block copolymer and a diol-functionalized polymer. At neutral pH, increasing glucose concentrations competitively disrupted these interactions, promoting micelle dissociation and insulin release. The complex micelles exhibited improved glucose responsiveness under physiologically relevant conditions compared with conventional phenylboronic acid-based micelles [126].

### 5.5. Hybrid and Multifunctional Nanoplatforms

Hybrid and multifunctional nanoplatforms integrate two or more material systems into a single carrier to combine the benefits of each constituent. In diabetes therapy, these platforms are valuable because they can enhance drug loading, stability, glucose responsiveness, and release control within one unified system. By incorporating polymers, lipids, inorganic materials, or bioactive molecules, hybrid systems can achieve more targeted and flexible antidiabetic drug delivery compared to single-component nanocarriers [127].

Various hybrid combinations have been investigated to optimize glucose-responsive insulin delivery, including (i) polymer–lipid hybrids, which strengthen insulin encapsulation and stability for oral delivery [128]; (ii) polymer–enzyme systems containing glucose oxidase, which enhance glucose sensitivity and promote accelerated insulin release upon triggering [129]; (iii) polymer–inorganic hybrids, such as mesoporous silica-based systems, which offer substantial loading capacity along with self-regulated release behavior [130]; and (iv) microgel–nanoparticle hybrid platforms, which have shown sustained glycemic control in diabetic models [131].

### 5.6. Comparative Performance of Glucose-Responsive Delivery Platforms

To facilitate comparison across different carrier architectures and sensing mechanisms, Table 7 summarizes the reported performance, preclinical evidence and principal translational limitations of representative glucose-responsive insulin-delivery platforms.

Overall, no platform currently combines rapid responsiveness, minimal basal leakage, complete reversibility, high insulin loading and prolonged glycemic control. GOx-based systems remain limited by oxygen dependence, enzyme instability and H_2_O_2_ generation, while PBA-based systems face concerns regarding physiological selectivity and competing biological diols; ConA-based systems additionally raise immunogenicity and safety concerns [67,94,101,103,124,131]. Inconsistent reporting also prevents direct comparison and highlights the need for standardized evaluation under physiologically relevant glucose conditions.

Having considered the principal sensing mechanisms and carrier architectures, the following section evaluates their therapeutic applications, preclinical performance, and routes of administration.

## 6. Therapeutic Applications and Recent Advances

### 6.1. Therapeutic Applications

Glucose-responsive nanomedicine has emerged as a highly promising strategy to improve the management of diabetes through the development of self-regulated drug delivery systems. Smart nanoplatforms such as these are capable of sensing fluctuations in blood glucose concentrations and subsequently releasing insulin as well as other therapeutic agents in a controlled manner.

**Figure 11:**
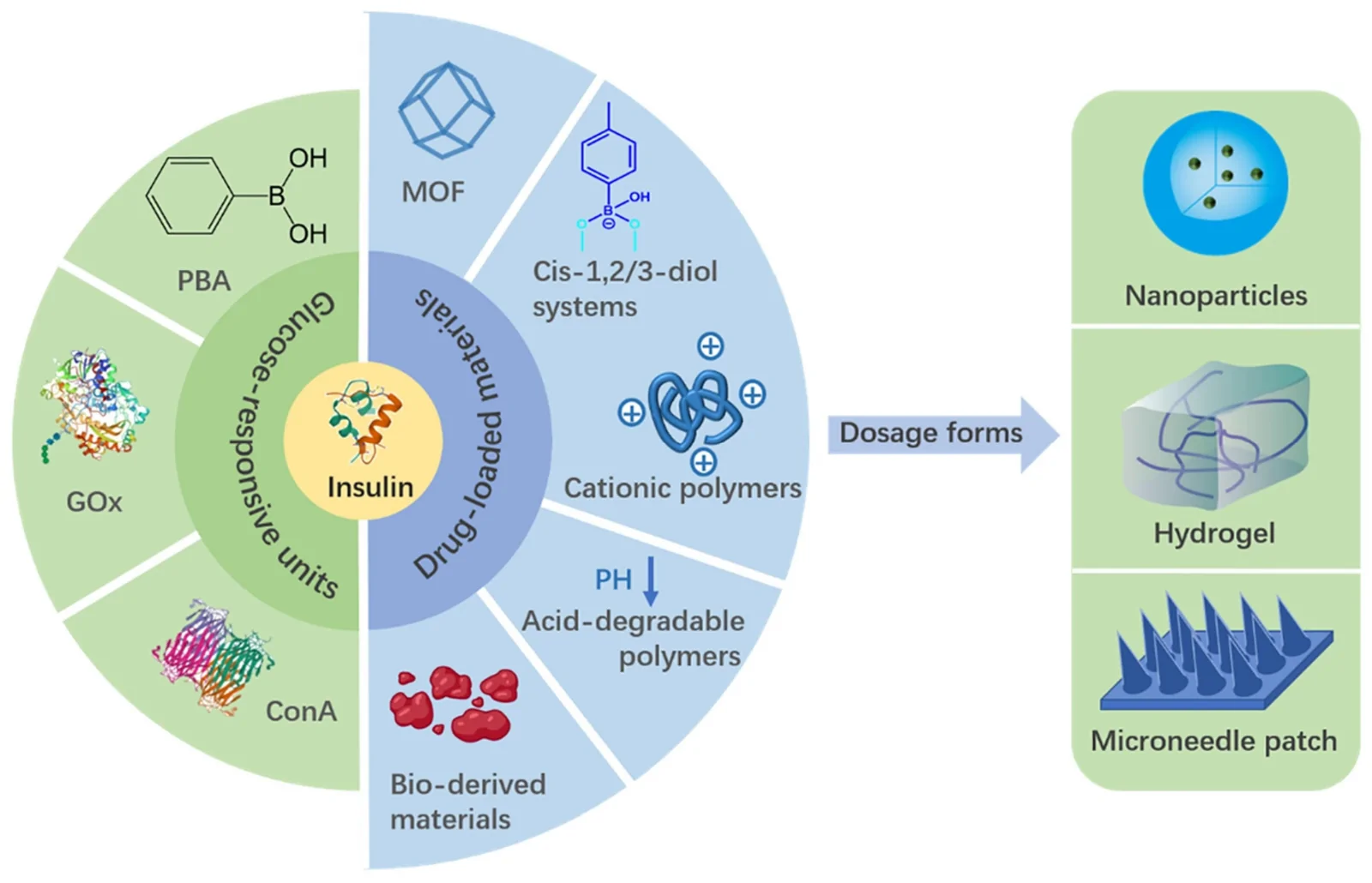
Overview of glucose-responsive insulin delivery systems, integrating glucose-responsive units, carrier materials, and principal dosage forms. Reprinted from Ref. [132].

Unlike conventional insulin therapy that often results in fluctuating glucose levels and poor adherence from patients, glucose-responsive systems aim to mimic physiological insulin secretion closely, thereby reducing the risk of hyperglycemia and hypoglycemia [132,133]. Recent advances in the field of nanotechnology have significantly improved the sensitivity, therapeutic efficiency, and design of such systems, making them increasingly relevant in diabetes therapy (Figure 11).

Insulin delivery remains the principal therapeutic application of glucose-responsive nanomedicine. Representative systems include GOx-containing hypoxia-sensitive vesicles, PBA-functionalized carriers, and hybrid GOx/PBA platforms, which have demonstrated glucose-triggered insulin release and glycemic regulation in preclinical models [132,134,135,136,137,138,139]. Their sensing mechanisms and carrier architectures are discussed in detail in Section 4 and Section 5. Beyond insulin delivery, glucose-responsive nanomedicine is being explored for the delivery of other antidiabetic agents, including glucagon-like peptide-1 (GLP-1) receptor agonists, gene-based therapeutics, and metformin [140]. The encapsulation of the agents mentioned within nanoscale carriers enhances bioavailability, improves drug stability, and protects against premature degradation. Nanocarriers can also facilitate targeted drug delivery to the pancreas or insulin-sensitive organs, therefore improving therapeutic outcomes, while simultaneously minimizing systemic side effects. Recent studies have further explored the use of nanotechnology for co-delivery strategies, in which insulin and other antidiabetic compounds are incorporated into a single glucose-responsive platform to achieve synergistic therapeutic effects [141].

### 6.2. Preclinical Performance Evaluation

In vitro and in vivo studies have demonstrated the significant therapeutic potential of glucose-responsive nanomedicine. In vitro investigations evaluated glucose-triggered release kinetics, biocompatibility, and nanoparticle stability as well as responsiveness under simulated physiological conditions [142]. Meanwhile, in vivo studies in diabetic animal models have shown improved glycemic control, reduced fluctuations of glucose, and prolonged therapeutic activity as well as decreased frequency of insulin administration [143]. Some nanoplatforms have demonstrated their ability to reduce inflammatory responses and oxidative stress associated with the progression of diabetes [144]. These findings highlight the potential of these systems to improve long-term diabetes outcomes and patient quality of life.

### 6.3. Administration Routes and Emerging Technologies

Recent innovations in the field of smart nanomedicine design have focused on enhancing safety, responsiveness, and integration with advanced technology in healthcare. Wearable glucose monitoring devices and closed-loop insulin delivery systems are increasingly being combined with glucose-responsive nanocarriers to create automated platforms for diabetes management [136]. Microneedle-based systems that incorporate glucose-sensitive nanoparticles have recently emerged as minimally invasive approaches that allow painless and continuous insulin administration [137,145]. Researchers are also exploring biomimetic nanomaterials and multifunctional hybrid nanoparticles that are capable of simultaneous glucose sensing, drug delivery, and real-time monitoring of blood glucose levels [141]. These developments all support the growing shift towards personalized medicine, especially in diabetes care.

Several challenges continue to arise, despite these advances, which limit the clinical translation of glucose-responsive nanomedicine. Long-term biocompatibility, reproducibility, and regulatory approvals remain significant barriers [135]. Furthermore, variations in delayed responsiveness and potential immunogenicity should be addressed before clinical implementation becomes feasible. Nevertheless, ongoing progress in materials, nanotechnology, and biomedical engineering continues to accelerate development towards safer and more effective glucose-responsive systems, which are expected to play a transformative role in the future of diabetes therapy by enabling adaptive, patient-centered treatment strategies [145]. These unresolved barriers and their implications for clinical translation are examined in detail in the following section.

## 7. Clinical Translation and Future Challenges

Nanomedicine research has rapidly expanded, spanning a broad spectrum of disease applications, including chronic conditions such as diabetes [146]. However, significant physiological and biological barriers continue to impede clinical translation, resulting in only a limited proportion of nanoformulations progressing to clinical application [146]. The multifactorial pathophysiology of diabetes mellitus coupled with the inherently complex physicochemical nature of nanomedicine systems results in various translational barriers that limit clinical implementation [147,148]. These limitations include biocompatibility, toxicity concerns, long-term safety, reproducibility, scalability, manufacturing limitations, regulatory barriers, and clinical translation.

### 7.1. Biocompatibility and Toxicity Concerns

**Figure 12:**
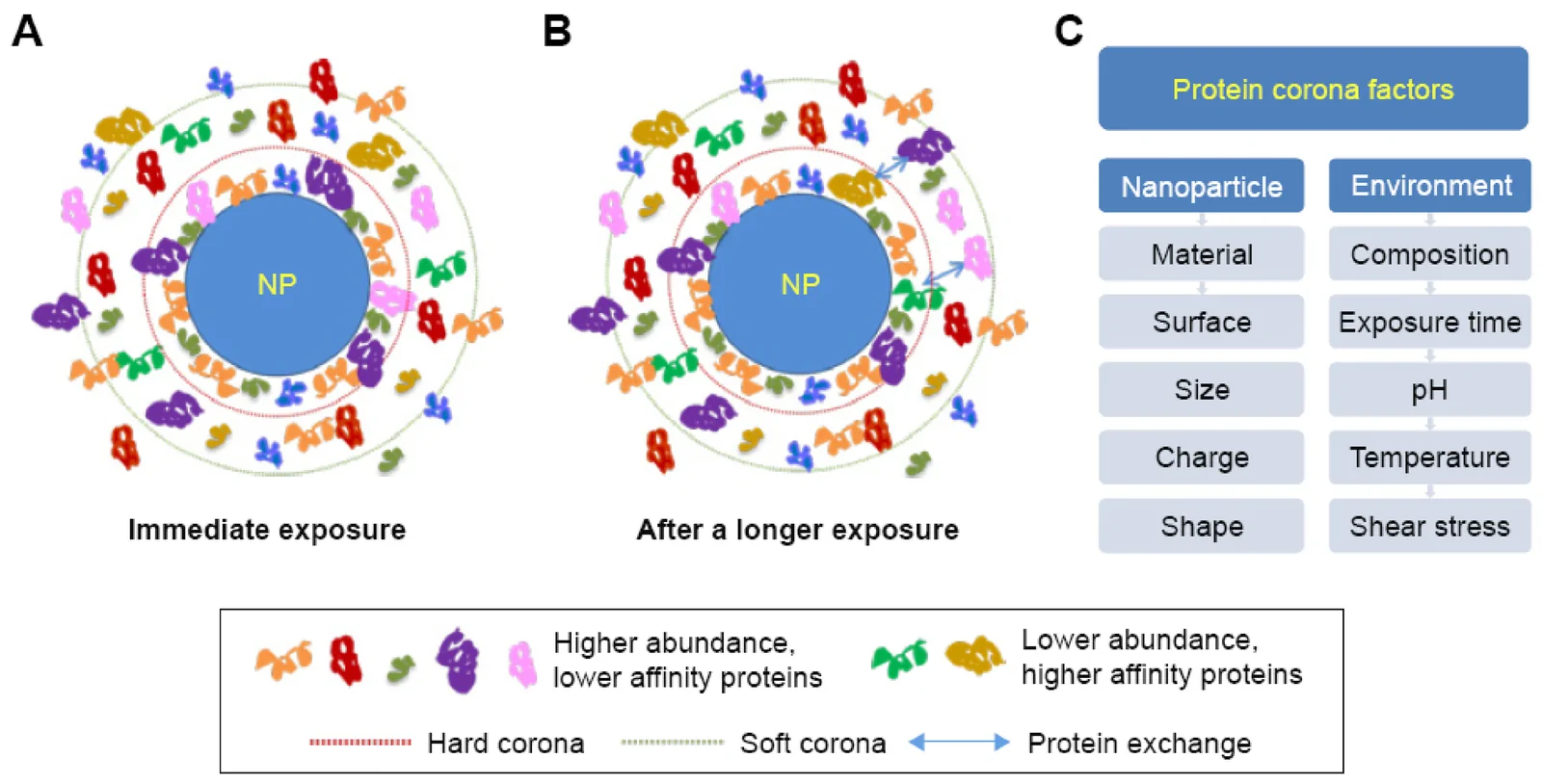
The formation of protein corona and exchange of adsorbed proteins over time in biological conditions. Notes: (**A**) Immediately upon exposure. (**B**) After a longer exposure time, with displacement of proteins among the hard corona, soft corona, and cellular environment. (**C**) Major factors affecting protein corona pattern divided into two categories: nanoparticle and environment. Abbreviation: NP, nanoparticle. Reprinted from Ref. [151].

For chronic conditions, such as diabetes mellitus, biocompatibility issues are even more pronounced due to prolonged and repeated exposure to nanomedicine therapies [149]. After entering biological fluids, nanoparticles rapidly adsorb proteins and other biomolecules, forming a dynamic protein corona that influences aggregation, cellular uptake, biodistribution, immune recognition and clearance. Opsonin-rich coronas may promote uptake by hepatic and splenic macrophages of the mononuclear phagocyte system, whereas dysopsonins may prolong circulation. Surface engineering can influence, but cannot completely control, corona composition because it varies with particle properties and the surrounding biological environment, as shown in Figure 12 [150,151,152]. Nanoparticles composed of silver can undergo surface-driven electron-transfer reactions and metal ion release, resulting in the generation of reactive oxygen species that decrease glutathione levels and increase reactive oxygen species (ROS) levels, eventually causing mitochondrial dysfunction and inflammation [153,154]. More recent studies have reported the protective effects of silver nanoparticles on pancreatic β cells, and accordingly, increasing attention has been given to utilizing their anti-inflammatory and antioxidant properties to improve insulin sensitivity and glycemic control, while mitigating the cytotoxic effects associated with systemic nanoparticle accumulation [154]. A subset of nanoparticles is engineered to target the endothelium for therapeutic delivery; however, their subsequent activation and increase in ROS result in nitric oxide depletion, exacerbating vascular inflammation and raising concerns about vascular toxicity [155].

### 7.2. Long-Term Safety and Reproducibility

**Figure 13:**
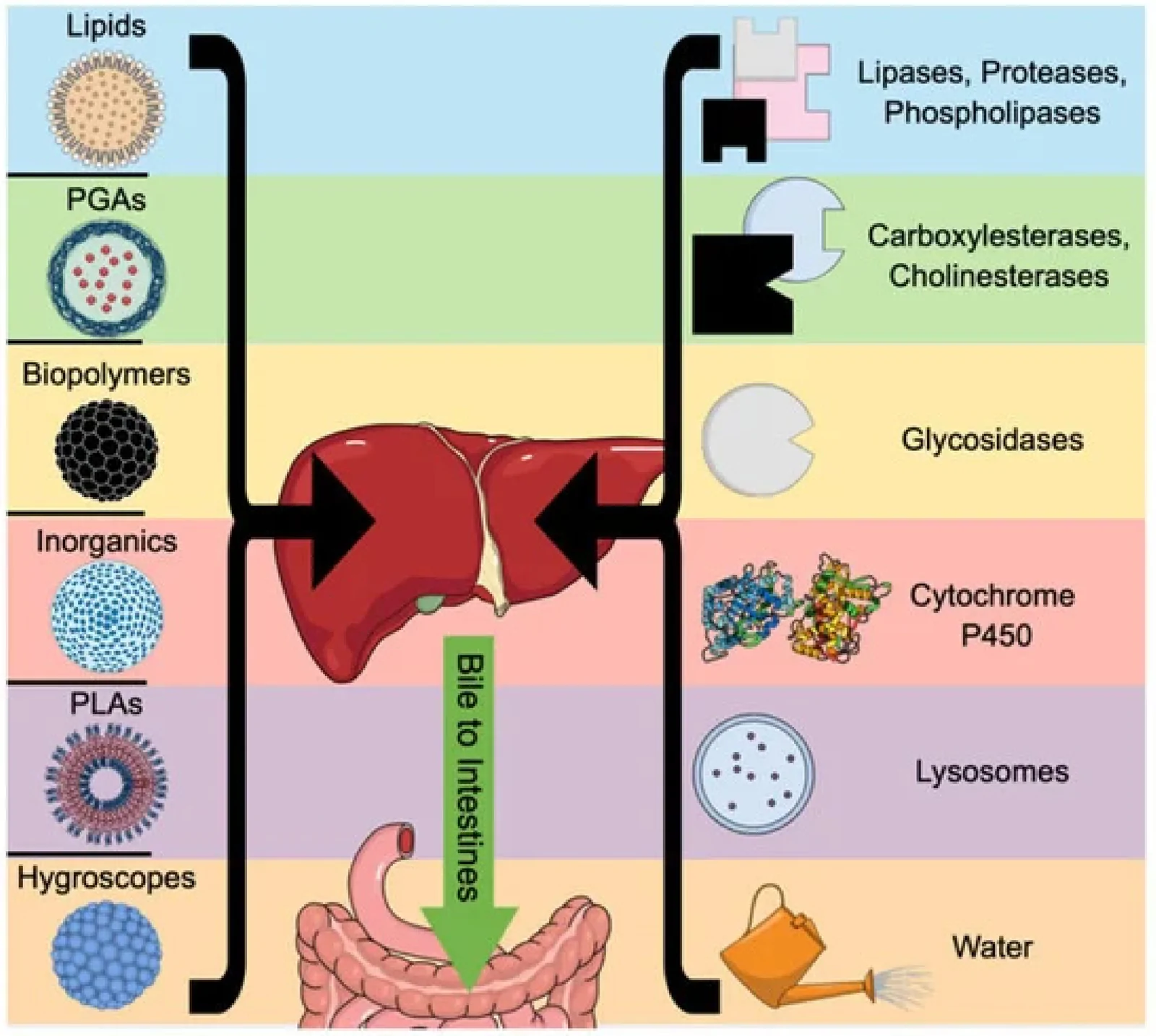
Molecular clearance: The liver contains diverse enzymes and an aqueous environment to metabolize nearly any nanoparticle at the molecular level, breaking down particles over time to incorporate them into bile for excretion. Black arrows indicate movement toward hepatic processing, whereas the green arrow indicates biliary excretion into the intestine. The colored bands associate the nanoparticle classes with their representative degradation or clearance mechanisms. PGAs include polyglutamic and polyglycolic acids; biopolymers include chitosan, dextran, and hyaluronic acids; inorganics include silica, gold, and carbon; PLAs include all polylactic acids; and hygroscopes include water-attracting compounds such as bile salts and wetting agents such as Tween. Reprinted from Ref. [157].

Nanoparticle disposition depends on size, shape, surface charge, composition, protein-corona formation and biodegradability. As summarized in Figure 13, clearance may involve material degradation, renal elimination, uptake by the mononuclear phagocyte system and hepatobiliary excretion. Small particles or degradation products may undergo renal elimination, whereas larger or opsonized particles are more commonly taken up by hepatic and splenic macrophages. These pathways do not guarantee complete clearance; slow degradation and repeated administration may lead to accumulation in the liver, spleen, kidneys or lungs [156,157]. Consequences of bioaccumulation include progressive organ burden, reduced detoxification, disturbance of normal physiological clearance systems, oxidative stress, and chronic inflammation [157,158]. At the molecular level, protein corona formation determines the biological identity and fate of the nanoparticle; however, even for biodegradable nanoparticle systems, degradation kinetics must be evaluated as incomplete breakdown can stimulate accumulation of residual material in organs [157]. For example, polymers such as PLGA degrade into lactic acid and glycolic acid; accumulation of these metabolites creates an acidic microenvironment that can trigger inflammatory signaling pathways and autocatalysis of PLGA, resulting in faster polymer degradation [159].

Reproducibility and batch-to-batch variability represent significant limitations in the production of nanoparticles, as minor deviations in synthesis conditions can result in significant nanoparticle property changes [158,159]. The lack of standardized physicochemical characterization in nanomedicine and the variability among experimental designs across nanomedicine studies highlight the reproducibility issues in nanomedicine. Minute changes in the preparation methodology or formulation conditions of nanoparticles can result in significant changes in zeta potential, morphology, drug loading, release kinetics, and polydispersity, consequently leading to batch-to-batch variability, hindering clinical translation [160].

Experimental studies of polydopamine nanoparticles further demonstrate that controlled oxidation conditions and polymerization time can modify critical quality attributes, including particle size, zeta potential, morphology, surface roughness, and functional surface accessibility [161,162].

### 7.3. Scale-Up and Manufacturing Limitations

As noted above, limited reproducibility and pronounced batch-to-batch variability remain critical challenges in nanomedicine implementation. Consequently, scalability is one of the most significant barriers limiting the clinical translation of glucose-responsive nanomedicine, as physicochemical conditions can be difficult to reproduce consistently within industrial manufacturing settings [163,164]. Glucose-responsive nanomedicine relies on multiple sequential production steps, including nanoprecipitation, self-assembly, surface functionalization, and ligand conjugation. The transition from laboratory-scale to industrial-scale nanomedicine production poses scalability challenges, as each unit operation or production step can independently influence critical quality attributes (CQAs), such as particle size, encapsulation efficiency, and stability [165]. Consequently, insufficient control at any step can introduce deviations that propagate through subsequent steps. Therefore, multistep manufacturing processes may allow deviations to accumulate across different stages, resulting in significant alterations in the final nanoparticle product. Transitioning from small-scale production to industrial manufacturing also presents significant cost burdens [166].

In addition to technical complexity, industrial manufacturing must comply with Good Manufacturing Practice (GMP) requirements while maintaining standardized nanoparticle physicochemical properties. Manufacturing standards and GMP quality guidelines are still evolving in the field of nanomedicine, and a universally standardized nanomedicine-specific regulatory framework has not yet been established [167]. Before translating laboratory-scale nanomedicine synthesis to large-scale industrial production, GMP-compliant pilot manufacturing processes should be established to support reproducible scale-up and the production of materials for clinical evaluation [168]. A concise summary of the barriers hindering the clinical translation of glucose-responsive nanomedicine is presented in Table 8.

### 7.4. Regulatory Barriers

Nanomedicines are regulated within existing medicinal-product frameworks, supplemented by nanomaterial- and product-specific guidance. The U.S. Food and Drug Administration (FDA) and the European Medicines Agency (EMA) apply established requirements for quality, safety and efficacy while requesting additional characterization and manufacturing data when nanoscale attributes affect product performance. However, no single harmonized international pathway exists, and data requirements may remain product- and jurisdiction-specific [168,169,170,171].

For example, Lipodox^®^, a liposomal doxorubicin product, was approved by the FDA as generic nanomedicine, whereas the EMA did not approve the product, claiming it did not demonstrate bioequivalence of free doxorubicin [170]. This is an example of how the same product can be interpreted and regulated differently across jurisdictions due to the lack of defined criteria, highlighting the need for harmonized global standards in nanomedicine implementation.

Moreover, to facilitate industrial manufacture, glucose-responsive nanomedicine systems must be stable during global transport and long-term storage [172]. Studies report that vesicular nanocarriers specifically are prone to aggregation secondary to Ostwald ripening, resulting in loss of uniformity and reduced stability [173]. Moreover, liposomes are composed of phospholipid bilayers, susceptible to oxidation and consequent liposomal breakdown, causing the encapsulated drug to leak out. Nanoparticles with low zeta potential demonstrate reduced repulsion and therefore are more likely to aggregate [174]. This nanoparticle aggregation can increase particle size, alter drug release kinetics, and reduce the stability of the nanoparticle, resulting in unpredictable drug delivery [175]. Stability limitations during storage, encompassing nanoparticle aggregation, unintended drug leakage, and alterations in drug release profiles, underscore the need for rigorous regulatory oversight to ensure product consistency and therapeutic efficacy [172,173,174,175,176,177].

### 7.5. Clinical Development Status and Translational Potential

Most glucose-responsive nanomedicine platforms remain at the in vitro or animal-study stage. Although GOx-, PBA-, and ConA-based nanocarriers have demonstrated glucose-triggered drug release and glycemic control in experimental models, their clinical safety and efficacy have not been established. MK-2640 is the most advanced glucose-responsive insulin to have undergone human testing; however, it is a soluble insulin-saccharide conjugate rather than a nanocarrier. In Phase 1 testing, MK-2640 was generally well tolerated but showed approximately 25-fold lower potency than regular human insulin and insufficient glucose-responsive performance [112]. Table 9 highlights the substantial gap between promising preclinical evidence and clinical development. Therefore, the clinical prospects of nanocarrier-based systems should be interpreted as future potential rather than evidence of clinical readiness.

Table 10 shows that several strategies have been proposed to overcome challenges related to protein corona formation, immune recognition, bioaccumulation, manufacturing reproducibility, scalability, regulatory uncertainty, and patient acceptance [178,179]. For example, studies have demonstrated the potential of PEGylation to reduce protein adsorption and suppress protein corona formation, subsequently improving nanoparticle stability in physiological fluids. Certain manufacturing protocols have been developed in accordance with GMP requirements, including microfluidic manufacturing protocols for mRNA vaccine lipid nanoparticles. These protocols specify controlled production steps, including defined microfluidic mixing ratios and laminar-flow mixing, followed by tangential-flow filtration [179]. Experimental studies have also evaluated autonomous chemical platforms that integrate artificial intelligence (AI) modules, particularly closed-loop optimization processes, into nanoparticle production to continuously adjust reaction conditions in real time. These studies demonstrated a highly reproducible nanoparticle production workflow that may improve scalability and consistency [180].

## 8. Conclusions and Future Perspectives

Glucose-responsive nanomedicine represents a significant advancement in diabetes therapy by enabling self-regulated, glucose-triggered delivery of insulin and antidiabetic agents. This review has highlighted the major glucose-sensing mechanisms, including glucose oxidase-based, phenylboronic acid-based, and lectin-mediated systems, along with diverse nanocarrier platforms such as polymeric nanoparticles, nanogels, liposomes, micelles, and hybrid nanostructures. These smart systems demonstrate superior glycemic control, enhanced drug stability, and improved patient adherence compared to conventional therapies in preclinical models.

However, significant barriers to clinical translation remain, including biocompatibility concerns, long-term safety validation, manufacturing scalability, and regulatory approval challenges. Future development should focus on rational design strategies, biocompatible biodegradable carriers, comprehensive clinical trials, and collaborative efforts between academia and industry to establish standardized manufacturing protocols. With continued advancement and systematic resolution of translational barriers, glucose-responsive nanomedicine has substantial potential to redefine diabetes management through intelligent, personalized therapeutic strategies that more closely mimic physiological insulin secretion and improve patient outcomes.

## Figures and Tables

**Table 1:** Adverse effects of oral hypoglycemic agents.

Drug Class	Adverse Effects	References
Biguanides	Gastrointestinal disturbances: nausea, diarrhea, abdominal discomfort Vitamin B12 deficiency	[39]
Thiazolidinediones	Fluid retention Peripheral edema Precipitating congestive heart failure Increased bone fracture risk	[40]
SGLT2 inhibitors	Urinary tract infections Genital fungal infections Euglycemic diabetic ketoacidosis Acute kidney injury	[41]

**Table 2:** Factors contributing to poor adherence and limitations of conventional insulin therapy [53].

Challenge	Clinical Impact	References
Multiple daily injections	Lipodystrophy Injection burden Treatment fatigue	[36,53]
Frequent glucose monitoring	Increased self-management complexity	[53,54,55,56]
Carbohydrate counting	Increased self-management complexity Cognitive burden	[43,53]
Dose adjustments	Risk of dosing error Increased treatment complexity Recurrent patient visits	[53]
Injection site complications	Lipodystrophy Reduced treatment adherence	[35,36,53,54,55,56]
Missed or delayed doses	Glycemic variability Poor glycemic control Microvascular and macrovascular complications	[32,43,50,54,55,56]

**Table 3:** GOx-mediated platforms [90].

Method	Platform	Drug Delivery Mechanism
Self-assembly	LbL films	Glucose-induced decomposition of films with insulin permeation
	Vesicles	Dissociation or destruction of vesicles induced by gluconic acid, H_2_O_2_, and hypoxia
Cross-linking	Hydrogels	Structural changes in response to pH changes in microenvironments, or acidic biodegradation of pH-sensitive materials
	Microgels	
Weak physical interaction	Mesoporous silica materials	Permeation changes in multilayers coated on mesoporous silica materials, or opening of pores on mesoporous silica materials due to the glucose-induced uncapping of gated materials
Fabrication	Devices with an insulin reservoir	Permeation changes in membrane used for sealing of insulin reservoir

Abbreviations: GOx, glucose oxidase; H_2_O_2_, hydrogen peroxide; LbL, layer-by-layer.

**Table 4:** Summary of research on PBA in glucose-responsive hydrogels in the past ten years [95].

Type of PBA	Materials	Function	Type of Hydrogel
3-aminomethyl phenylboronic acid	Hyaluronic acid; polyethylene glycol diacrylates; myricetin	Hydrogel has exhibited great potential for diabetic wound treatment	Hydrogel dressings
3-aminomethyl phenylboronic acid	Hyaluronic acid methacrylate; phenylboronic acid; hyaluronic acid derivative; catechin	Hydrogel with potential for application in diabetic wound treatment	Hydrogel dressings
Phenylboronic acid	Folliculin-interacting protein 1; hyaluronic acid; phenylboronic acid; fulvic acid	Promising hydrogel strategy for chronic diabetic wound repair	Hydrogel dressings
4-(Bromomethyl)-Phenylboronic acid	Gallic acid; chitosan; poly (ethylene glycol) diacrylate; polyethyleneimine; phenylboronic acid	Hydrogel with glucose-responsive hyperglycemia regulation and antioxidant activity for enhanced diabetic wound repair	Hydrogel dressings
Formylphenylboronic acid	Polyvinyl alcohol; nature-abundant proteins (bovine serum albumin, egg albumin, casein); formylphenylboronic acid	Injectable and potential as smart insulin for in vivo applications shortly	Injectable hydrogel
3-Fluoro-4-carboxy-phenylboronic acid	3-fluoro-4-carboxy-phenylboronic acid-grafted polylysine; natural guar gum	Maintaining performance via glucose-responsive transdermal insulin delivery	Hydrogel dressings
3-Fluoro-4-carboxy-phenylboronic acid	Galactosyl; 3-fluoro-4-carboxy phenylboronic acid	Hydrogels that increase the crosslinking density can slow the spread of insulin in the body and control the release of insulin	Injectable hydrogel
4-(2-Acrylamidoethylcarbamoyl)-3-fluoro-phenylboronic acid	Biocompatible silk fibroin (SF); 4-(2-acrylamidoethylcarbamoyl)-3-fluorophenylboronic acid; acrylamide	Regulates the epidermal layer and releases insulin autonomously, corresponding to the glucose change pattern	Hydrogel dressings
3-(Acrylamido)phenylboronic acid	N-isopropylacrylamide; 3-(Acrylamido) phenylboronic acid; Alginate	In response to changes in glucose concentration, reversible sol–gel conversion is generated to achieve self-regulated release of insulin	Hydrogel dressings
3-(Acrylamido)phenylboronic acid	N-isopropylacrylamide 99% stabilized; 2-(Dimethylamino) ethyl methacrylate; 3-(Acrylamido)phenylboronic acid	pH-dependent insulin release patterns	Hydrogel
4-Carboxy-3-fluorophenylboronic acid	2,5-Dimethylbenzoic acid; N-(2-Hydroxyethyl) maleimide; 3-Pyridylboronic acid, 2-(Bromomethyl)benzoic acid, 3-(Bromomethyl)benzoic acid, Hydroxybenzotriazole monohydrate; PEG	Improved responsiveness translates to more rapid blood glucose correction in a rodent diabetes model	Injectable hydrogel
4-Carboxy-3-fluorophenylboronic acid pinacol ester	4-Armpolyethylene glycol; 4-Carboxy-3-fluorophenylboronic acid pinacol ester	Accelerate the release of insulin to glucose	Injectable hydrogel
4-Carboxyphenylboric acid	Quaternary ammonium chitosan; Dihydrocaffeic acid; l-arginine; oxidized hyaluronic acid-dopamine; methacrylated poly (vinyl alcohol) (methacrylated PVA); phenylboronic acid; gallium porphyrin; 3-Amino-1,2 propanediol; Insulin	Excellent biocompatibility, slow drug release	Hydrogel
Carboxy phenylboronic acid	Natural silk fibroin protein; carboxy phenylboronic acid	Modification of silk fibroin into a glucose-responsive hydrogel platform for regulated and functional insulin delivery application	Injectable hydrogel
4-Vinyl-phenylboronic acid	Intelligent cellulose; 4-Vinyl-phenylboronic acid	The complex hydrogel self-regulates insulin release under different concentrations of glucose	Hydrogel
3-Acrylamidophenylboronic acid	Acrylamide or N-isopropylacrylamide;N,N′-methylenebisacrylamide; 3-acrylamidophenylboronic acid; N-(3-dimethylaminopropyl)acrylamide	Method for determining the amount of bound glucose in hydrogels	Hydrogel

Abbreviations: PBA, phenylboronic acid; PEG, poly(ethylene glycol); PVA, poly(vinyl alcohol); SF, silk fibroin.

**Table 5:** Representative examples of dual-responsive injectable hydrogels for insulin delivery [83].

Dual-Responsiveness	Polymer	Biocompatibility	Insulin Loading Capacity (LC) or Encapsulation Efficiency (EE)	In Vitro Insulin Release (Duration and Cumulative Release Percentage)	Duration of Glycemic Control In Vivo After Single Injection
Glucose and Temperature	Alginate-g-P(NIPAM-co-AAPBA)	Viability of L929 mouse fibroblasts remained at 100% after incubation for 24 h	Loading ratio 1.0 g/L	48 h; 70% at 27.8 mmol/L glucose condition (GC) and 30% at 5.6 mmol/L GC	NR
	F127-PBA	Viability of C2C12 cells was maintained at over 95% after incubation for 24 h	Loading ratio 20 μg/100 μL	8 h; 36% at 0 mmol/L GC and 53% at 22.2 mmol/L GC	9 h in mice
	P(Lys-co-LysFCPBA)-b-PEG-b-P(Lys-co-LysFCPBA) & γ-P(GA-co-GAGal)	No inflammation at mice’s injection sites after 14 d	LC: 8.6 ± 0.4 wt%; EE: 13.0 ± 0.2 wt%	12 h; 62.6% at 27.8 mmol/L GC and 17.7% at 5.6 mmol/L GC	24 h in mice
Glucose and pH	CSPBA/PEGCHO/PVA/GOx	HSF cells exhibited higher viability, and injection sites on mice showed no inflammation after 4 weeks	LC: 0.3%	36 h; 16% at pH 7.4, and 70.2% at pH 6.5; 33% at 5.6 mmol/L GC and 51.8% at 16.7 mmol/L GC	11 d in mice
Temperature and pH	OS-b-PCL-b-PEG-b-PCL-b-OS	No abnormal symptoms at the injection site of mice after 1 month	LC: 20%; EE: 95.85%	30 d; Over 80% at pH 7.4	NR
	OS-b-PLA-b-PEG-b-PLA-b-OS	The viability of 293 T and RAW 264.7 cells remained over 80% after incubation for 24 h	LC: 20%; EE: 96%	NR	60 h in mice

Abbreviations and polymer notation: AAPBA, 3-acrylamidophenylboronic acid; CSPBA, phenylboronic acid-modified chitosan; EE, encapsulation efficiency; F127, Pluronic F127 (poloxamer 407); FCPBA, 4-carboxy-3-fluorophenylboronic acid; GA, glutamic acid; GAGal, galactose-modified glutamic acid; GC, glucose concentration; GOx, glucose oxidase; HSF, human skin fibroblasts; LC, loading capacity; Lys, lysine; NIPAM, *N*-isopropylacrylamide; NR, not reported; OS, oligo-serine; PBA, phenylboronic acid; PCL, poly(ε-caprolactone); PEG, poly(ethylene glycol); PEGCHO, benzaldehyde-capped poly(ethylene glycol); PLA, poly(lactic acid); PVA, poly(vinyl alcohol); b, block; co, copolymer; g, graft; γ, gamma linkage.

**Table 6:** Comparative analysis of the major glucose-sensing mechanisms used in glucose-responsive nanomedicine.

Glucose-Sensing Mechanism	Principal Strength	Key Translational Challenge	Clinical Implication	Ref.
Glucose oxidase (GOx)	High glucose sensitivity and efficient glucose-triggered insulin release	Oxygen dependence, hydrogen peroxide generation, and enzyme instability may affect long-term performance	Requires strategies that improve enzyme stability and reduce oxidative stress before wider clinical translation	[83,84,87,90]
Phenylboronic acid (PBA)	Protein-free glucose recognition with high chemical stability and flexible chemical design	Glucose binding under physiological conditions requires optimization of boronic acid chemistry	Modified PBA derivatives may improve physiological glucose responsiveness while maintaining stability	[91,92,94,106]
Concanavalin A (ConA)	Strong and reversible glucose-binding capability	Immunogenicity and toxicity remain the major barriers to clinical application	Biocompatibility rather than sensing performance currently limits clinical translation	[96,97,98]
Hybrid glucose-sensing systems	Combine complementary mechanisms to improve responsiveness while reducing limitations of individual systems	Greater formulation complexity and manufacturing challenges	Hybrid platforms may provide a better balance between sensitivity, stability and safety but require further optimization for large-scale production	[107,108,110]

Abbreviations: ConA, concanavalin A; GOx, glucose oxidase; PBA, phenylboronic acid.

**Table 7:** Comparative performance of representative glucose-responsive insulin-delivery platforms.

Platform and Mechanism	Glucose-Response Performance and Loading	Administration and Evidence	Main Translational Limitation	Ref.
Hypoxia- and H_2_O_2_-sensitive polymersome microneedles; GOx-based	Tested at 0, 100 and 400 mg/dL; response within 1 h; little basal leakage; pulsatile release but irreversible vesicle disassembly; LC: 3.2%	Transcutaneous; STZ-diabetic mice; glycemic control for approximately 6 h, with activity up to 10 h	Low loading, oxygen and enzyme dependence, and microneedle scale-up	[103]
POSS–APBA nanomicelles; PBA–glucose binding	Tested at 0–375 mg/dL; release began within 10 min and equilibrated within approximately 30 min; no release without glucose; reversible; EE: 73.2%, LC: 50.5%	In vitro release and cellular biocompatibility testing; no in vivo evaluation	Competing biological diols, broad particle-size distribution and absence of animal validation	[94]
ConA-loaded chitosan–Pluronic hydrogel; glucose–ConA affinity	Tested at 400 and 1000 mg/dL; 97% release over 7 days; normoglycemic response and repeated cycling not established; loading efficiency: 46.8%	In vitro RIN-5F cell model; no in vivo evaluation	Supraphysiological glucose conditions, ConA leakage and immunogenicity	[101]
Multivesicular liposomes; combined PBA and GOx response	Pulsatile release at alternating 100 and 400 mg/dL; approximately 2 h recovery after glucose challenge; membrane disruption irreversible; loading NR	Injectable; chemically induced type 1 diabetic rats; normoglycemia achieved, duration NR	Complex formulation, enzyme instability and H_2_O_2_ generation	[124]
Acetylated-dextran nanoparticles within alginate microgels; GOx/CAT response	Responded to normoglycemic and 400 mg/dL conditions; reduced premature leakage and supported repeated glucose challenges; nanoparticle degradation irreversible; loading NR	Subcutaneous; diabetic mice; glycemic control up to 22 days after two doses of 60 IU/kg	High insulin dose, repeated administration and lack of large-animal validation	[131]
Oral polymer–insulin complex forming worm-like micelles; glucose-dependent charge switching	Rapid release under hyperglycemia with limited low-glucose activity; standardized range, response time and LC NR	Oral; mice and diabetic pigs; significant glucose reduction with limited hypoglycemia, duration NR	Gastrointestinal variability, polymer safety and preclinical development stage	[67]

Notes: Glucose concentrations represent experimental testing conditions, not validated clinical thresholds. In vitro and in vivo response times should not be directly compared. NR indicates that a directly comparable value was not reported and was not estimated. Abbreviations: APBA, aminophenylboronic acid; CAT, catalase; ConA, concanavalin A; EE, encapsulation efficiency; GOx, glucose oxidase; LC, loading capacity; NR, not reported; PBA, phenylboronic acid; POSS, polyhedral oligomeric silsesquioxane; STZ, streptozotocin.

**Table 8:** Summary of major barriers limiting clinical translation of glucose-responsive nanomedicine.

Barrier	Underlying Issue	Clinical Impact	References
Immune clearance	Macrophage uptake by MPS	Short circulation time	[150,151,152]
Long-term toxicity	Organ accumulation and ROS generation	Safety issues	[153,154]
Protein corona formation	Altered biological identity	Reduced targeting efficiency	[157]
Reproducibility	Batch-to-batch variability	Inconsistent efficiency	[158,159]
Manufacturing scalability	Complex multistep production	High production costs	[165]
Regulatory barriers	Limited international harmonization and product-specific regulatory requirements	Delayed approval	[168,169,170,171]

Abbreviations: MPS, mononuclear phagocyte system; ROS, reactive oxygen species.

**Table 9:** Clinical development status of glucose-responsive therapeutic systems.

Platform	Development Stage	Main Outcome	Main Limitation	Ref.
GOx-, PBA-, and ConA-based nanocarriers	In vitro and preclinical animal studies	Glucose-triggered release and glycemic control demonstrated experimentally	No established human safety or efficacy; mechanism-specific stability and safety concerns	[87,94,101,103]
MK-2640 insulin-saccharide conjugate	Phase 1; NCT02269735	Generally well tolerated but approximately 25-fold less potent than regular human insulin	Insufficient clinical glucose responsiveness and reduced potency	[112]

**Table 10:** Future strategies to facilitate clinical translation of glucose-responsive nanomedicine.

Challenge	Proposed Solution	References
Bioaccumulation	Biodegradable materials	[157,158]
Protein corona formation	Surface engineering and stealth coatings	[178]
Immune recognition	PEGylation and biomimetic nanoparticles	[178]
Reproducibility	Standardized manufacturing protocols	[178]
Scalability limitations	Continuous-flow and automated production, AI machine learning	[178]
Regulatory uncertainty	Harmonized regulatory frameworks	[179,180]

## Data Availability

No new data were created or analyzed in this study. Data sharing is not applicable to this article.
